# Discovery of a new bacterium, *Microbacterium betulae* sp. nov., in birch wood associated with hypersensitivity pneumonitis in woodworkers

**DOI:** 10.1111/1758-2229.13311

**Published:** 2024-08-12

**Authors:** Mariola Paściak, Krzysztof J. Pawlik, Dariusz Martynowski, Łukasz Łaczmański, Jarosław Ciekot, Bogumiła Szponar, Angelina Wójcik‐Fatla, Barbara Mackiewicz, Ewelina Farian, Grażyna Cholewa, Alicja Cholewa, Jacek Dutkiewicz

**Affiliations:** ^1^ Hirszfeld Institute of Immunology and Experimental Therapy, Polish Academy of Sciences Wrocław Poland; ^2^ Department of Health Biohazards and Parasitology Institute of Rural Health Lublin Poland; ^3^ Department of Pneumonology, Oncology and Allergology Medical University Lublin Poland

## Abstract

A Gram‐positive, aerobic, rod‐shaped mesophilic bacterium was isolated from birch wood, referred to as the AB strain. Allergological tests suggest that this strain may cause allergic alveolitis in sawmill workers. Employing a polyphasic taxonomic approach, the AB strain's 16S rRNA gene sequence showed high similarity to *Microbacterium barkeri* and *M. oryzae*, with 97.25% and 96.91%, respectively, a finding supported by *rpoB* and *gyrB* sequence analysis. Further genome sequence comparison with the closely related *M. barkeri* type strain indicated a digital DNA–DNA hybridization value of 25.5% and an average nucleotide identity of 82.52%. The AB strain's cell wall peptidoglycan contains ornithine, and its polar lipids comprise diphosphatidylglycerol, phosphatidylglycerol, and unidentified glycolipids. Its major fatty acids include anteiso C15:0, anteiso C17:0, and iso C16:0, while MK‐10 is its predominant respiratory quinone. Comprehensive analysis through 16S rRNA, whole‐genome sequencing, phenotyping, chemotaxonomy, and MALDI‐TOF MS profiling indicates that the AB strain represents a new species within the *Microbacterium* genus. It has been proposed to name this species *Microbacterium betulae* sp. nov., with AB^T^ (PCM 3040^T^ = CEST 30706^T^) designated as the type strain.

## Graphical abstract

Through the use of 16S rRNA and whole‐genome sequence analysis, along with phenotyping, chemotaxonomy, and MALDI‐TOF MS profiling, a strain isolated from birch wood has been identified as a new species. This novel species falls within the *Microbacterium* genus and has been proposed to be named *Microbacterium betulae* sp. nov.

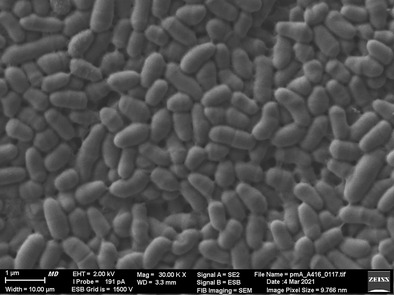

## INTRODUCTION

Hypersensitivity pneumonitis (HP) is an immune‐mediated syndrome that often goes undiagnosed (approximately 30–60% of cases) due to challenges in identifying the causative agents, which can include bacteria, fungi, mycobacteria, animal and plant proteins, chemicals, and metals. Antigenic diversity among bacterial taxa related to the rural environment and responsible, e.g., for farmer's lung disease contains mainly thermophilic actinomycetes species, but also species such as *Pantoea agglomerans* (previously *Erwinia herbicola*), *Bacillus* spp., *Acinetobacter* spp., and *Achromobacter* spp. (Nogueira et al., 2019; Quirce et al., 2016; Calaras et al., 2024). Regarding woodworkers, *Pantoea agglomerans* have been identified as a potential causative agent of respiratory diseases (Wójcik‐Fatla et al., 2022), similar to bacteria from the *Microbacterium* genus, initially classified as *M. barkeri*. In the same study (2017–2019), a diagnosis of HP was established in 5 sawmill workers from Lublin Province (eastern Poland) who were exposed to the inhalation of dust from white warty birch (*Betula pendula*) and subsequently treated at the Medical University in Lublin (Mackiewicz et al., 2019).

For the identification of pathogenic allergens associated with causing disease symptoms, the cylindrical blocks were cut from transverse sections of birch logs and provided to the Institute of Rural Health in Lublin for microbiological examination. The samples were taken with the original drilling device patented in the USA, which collects pulverized wood into a flask attached beneath the bit in a one‐step sterile process (Dutkiewicz et al., 1989). The concentration and composition of bacteria were determined by dilution plating on tryptic soya agar (TSA). The results depended on the sampling site on the wood block section, which showed two distinct rings: central brownish and peripheral yellowish rings. The content of microorganisms in the peripheral ring was approximately 10,000 greater than that in the central ring (Mackiewicz et al., 2019). This could be explained by the fact that the peripheral ring corresponded to the ‘sapwood’ (phloem) tissue, which transports photosynthetic products of high nutritional value that are attractive to bacteria, while the central ring corresponded to the ‘heartwood’ (xylem), which consists mainly of cells that transport less attractive water.

The most numerous bacterial strains were the Gram‐positive coryneform AB strain, whose concentration in the wood of the peripheral ring was 1.8 × 10^8^ CFU g^−1^ DCW (dry cell weight), followed by the Gram‐negative strain *Pantoea agglomerans* (1.6 × 10^8^ CFU g^−1^ DCW). Preliminary studies based on the 16S rRNA gene sequence of only the prevailing coryneform isolate showed the highest similarity (97.5%) to that of *Microbacterium barkeri* DSM 20145 (GenBank accession no. X77446), which was originally described under this name.

This study presents a comprehensive taxonomic evaluation of an AB strain isolated from birch wood. The strain is suspected of causing HP in sawmill workers. Phenotypic, chemotaxonomic, and phylogenetic analyses revealed that this strain is a member of the *Microbacterium* genus and represents a novel species. The genus *Microbacterium* represents the Actinobacteria phylum and comprises approximately 132 validly published species (https://lpsn.dsmz.de/genus/microbacterium, accessed 24‐04‐2023). These bacteria were isolated from a wide variety of environments, including terrestrial‐, aquatic‐, and plant‐associated environments (Alves et al., 2014). Moreover, they were isolated from human blood, i.e., *M. ihumii* and *M. binotii* (Clermont et al., 2009; Yacouba et al., 2022), and from a cystic fibrosis patient, *M. yannicii* (Sharma et al., 2012), which indicates the pathogenic potential of these microorganisms.

## EXPERIMENTAL PROCEDURES

### Isolation and cultivation

Strain AB was isolated from a birch wood sample located in Bełżec, Lublin Province, Poland (coordinates, latitude: N: 50°22′18.944″, longitude: E: 23°27′17.507″). One hundred milligrams of the pulverized birch wood sample was suspended in 10 mL of sterile saline containing 0.1% Tween 80 (Sigma‐Aldrich, Germany) (v/v), and after vigorous shaking, serial dilutions in saline were made. The 0.1 mL aliquots of each dilution were spread on duplicate sets of blood (BL, Biomaxima, Poland) and TSA plates (Biomaxima, Poland) and incubated for one day at 37°C, 3 days at 22°C and 3 days at 4°C. The strain was cultivated on tryptic soya broth/agar (TSB/TSA, Biomaxima, Poland) or nutrient agar (NA, Biomaxima, Poland) at 28°C for 24–48 h unless otherwise stated.

The preliminary identification of the strain was confirmed by biochemical methods using the ENTEROtest 24 N (Erba Lachema, Czech Republic) and GEN III MicroLog M System (Biolog, Inc., USA) tests. As a positive control, the previously isolated *Pantoea agglomerans* strain from birch wood (Wójcik‐Fatla et al., 2022) was used. The strain has been deposited at the Polish Collection of Microorganisms in the Institute of Immunology and Experimental Therapy of the Polish Academy of Science in Wroclaw (Poland) under the number PCM 3041. The sequence of the 16S rRNA gene fragment has been deposited in GenBank under accession number MW647906 (*P. agglomerans* strain PCM 3041 16S ribosomal RNA gene, partial sequence).

### Phenotypic tests

Phenotypic tests were performed on the AB strain, in comparison to the type strains of the related *Microbacterium* spp: *M. barkeri* type strain DSM 20145, *M. sediminis* type strain DSM 23767 (Yu et al., 2013), *M. ulmi* type strain DSM 16931 (Rivas et al., 2004). Type strains were obtained from the German Collection of Microorganisms (DSMZ). *Microbacterium ulmi* was chosen because of a similar source (sawdust from *Ulmus nigra*).

Phenotypic tests were performed to determine the microscopic appearance, growth abilities, and metabolic traits of the selected *Microbacterium* strains. The microscopy tests included phase‐contrast microscopy (Gram staining, motility observation in the hanging drop) and electron microscopy. The growth tests were performed with TSA. They included the determination of the temperature and pH ranges that enabled bacterial growth as well as the determination of the highest concentration of NaCl (POCH, Poland) at which bacterial growth still occurred.

The breakdown of various compounds and the presence of different enzymes were determined by the use of the ENTEROtest 24 N (Erba‐Lachema, Czech Republic) and API ZYM systems (BioMérieux, France), respectively, whereas the carbon source utilization and chemical sensitivity were determined by the GEN III MicroLog M System (Biolog Inc., USA) test panel, according to the manufacturer's instructions. Catalase activity was examined by using a 3% H_2_O_2_ solution (Sigma‐Aldrich, Germany).

### Scanning electron microscopy

The cellular morphology of strain AB was assessed as previously described (Saeed et al., 2018) by scanning electron microscopy (SEM) with an Auriga 60 (Zeiss, Germany).

### Chemotaxonomic characterization

Biomass for chemotaxonomic studies was prepared by growing the strain AB and *M. barkeri* DSM 20145^T^, *M. sediminis* DSM 23767^T^, and *M. ulmi* DSM 16931^T^ in TSB broth for 48 h at 28°C. The cells were centrifuged (Heraeus Biofuge Stratos, Germany) at 4000x *g* for 20 min at 4°C, washed twice with PBS (phosphate‐buffered saline, pH 7.2–7.4) and Milli‐Q water, and then freeze‐dried.

The fatty acid profile was determined according to the GC techniques described by Sasser (Sasser, 2001) and Kämpfer & Kroppenstedt (Kämpfer & Kroppenstedt, 1996). Fatty acids were analysed by GC–MS on a Focus GC connected to an Ion Trap ITQ 700 with a Zebron ZB‐5HT (30 m × 0.25 mm × 0.25 μm) column (Phenomenex) with a temperature program of 150–270°C at 12°C/min in triplicate. The fatty acid profiles of strain AB were determined in parallel with those of *M. barkeri* DSM 20145^T^, *M. sediminis* DSM 23767^T^, and *M. ulmi* DSM 16931^T^ in this study. The alkali procedure for preparing mycolic acid methyl esters was used to detect mycolic acids (Embley & Wait, 1994).

The polar lipids of strain AB were analysed in parallel with those of *M. barkeri*, *M. sediminis*, and *M. ulmi*. Polar lipids were extracted and identified by two‐dimensional TLC (HPTLC silica gel 60, 10 x 10 cm, glass plates, Merck‐Millipore, Germany) according to the method of Minnikin et al. (Minnikin et al., 1984). The HPTLC plates were sprayed with specific reagents for total lipids (phosphomolybdic acid), phospholipids (Dittmer and Lester reagent), and glycolipids (orcinol reagent) (Mordarska & Paściak, 1994).

To determine the peptidoglycan structure, a purified cell wall was prepared according to a previously described method (Lechevalier & Lechevalier, 1970; Lechevalier & Lechevalier, 1980). Amino acids and peptides in the cell wall hydrolysates were examined by TLC as described by Schleifer (Schleifer, 1985). Subsequently, GC–MS analysis of chloroformate derivatives of amino acids (Badawy et al., 2008) was performed to determine the amino acid contents. Derivatives were prepared using the Phenomenex EZ: Faast Amino Acid Analysis Kit. GC–MS analysis was performed on a Focus GC connected with an Ion Trap ITQ 700, employing a ZB‐5HT capillary column and a temperature program of 110–320°C at 15°C/min, with helium as the carrier gas and a split ratio of 1:10. Whole cell wall sugars were analysed according to the methods of Lechevalier & Lechevalier (Lechevalier & Lechevalier, 1980; Lechevalier & Moss, 1977) and analysed by GC–MS under the same conditions as those for the fatty acids.

Isoprenoid quinones were extracted from wet cells according to Xie et al. (Xie, Pei, et al., 2021), purified by TLC and subsequently analysed by HPLC. A Thermo Scientific Ultimate 3000 HPLC equipped with an LPG‐3400SD pump, a WPS‐3000TSL autosampler, a TCC‐300 column compartment, and an FLD‐3400RS 2 PMT fluorescence detector (Thermo Scientific) were used. The chromatography column used was an Accucore C18 column (80 A, 2.6 μm, 100 × 2.1 mm, Thermo Scientific) with postcolumn reduction Bischoff 50 × 4,6 mm with zinc. The mobile phase was methanol: ethyl alcohol (70:30, v/v), the flow rate was 0.2 mL/min, and the eluent was monitored with an EX 220 nm EM 436 nm fluorescence detector. Data acquisition and processing were carried out with Chromeleon 7.2.6 software. MS analysis of menaquinones was performed on a Dionex 3000 RS‐HPLC instrument equipped with a DGP‐3600 pump, a WPS‐3000 TLS TRS autosampler, a TCC‐3000 RS column compartment, and a Bruker micrOTOF‐QII mass spectrometer detector (Bruker Daltonics, Germany). Data acquisition was carried out with HyStar software, and data processing was carried out with DataAnalysis 5.0 software.

### MALDI‐TOF MS

Matrix‐assisted laser desorption/ionization‐time‐of‐flight (MALDI‐TOF) mass spectra were generated from fresh cultures of strains AB, *M. barkeri* DSM 20145^T^, *M. sediminis* DSM 23767^T^, and *M. ulmi* DSM 16931^T^ grown aerobically on tryptic soy agar (TSA), nutrient agar (NA), and blood agar (BL) for 48 h at 30°C. Samples were prepared according to the formic acid/acetonitrile extraction method as described previously (Paściak et al., 2015), and by the direct smear method, i.e., bacterial colonies from TSA were applied directly on the MALDI target plate (MTP 384 polished steel BC, Bruker, USA), then air dried and overlaid with 70% formic acid and finally coated with the matrix solution. As a matrix, alpha‐cyano‐4‐hydroxycinnamic acid (HCCA) was used. The spectra were externally calibrated using the *E. coli* DH5‐alpha standard (Bruker Daltonics, Germany). Measurements and data processing were performed using the Ultraflextreme instrument, Bruker Biotyper version 3.1 (Bruker Daltonics), and ClinPro Tools version 3.0. The Biotyper database containing 8469 entries was used for strain identification. A dendrogram based on the similarity matrix was created using MALDI Biotyper 3.0 (Bruker).

### Sequencing of the 16S rRNA, *rpoB* and *gyrB* genes

Genomic DNA for sequencing of the 16S rRNA, *rpoB*, and *gyrB* genes was extracted using Genomic Mini AX Streptomyces (A&A Biotechnology, Poland). The 16S rRNA gene was PCR amplified using the universal oligonucleotide primers 16S start (AGAGTTTGATCMTGGCTCAG) and 16S stop (AAGGAGGTGWTCCARCC) as described by Paściak et al. (Paściak et al., 2014). The 16S rRNA amplicons were purified and then commercially sequenced in triplicate by Genomed Poland. Gene fragment amplification of the housekeeping genes was performed using the rpoBF (AAGGGMACSTTCGTCATAA) and rpoBR (CGATCAGACCGATGTTCGGG) primers for the *rpoB* gene and the gyrBF (GASSGCSTTCCTSAACAAGG) and gyrBR (GCNCGGAASCCCTCYTCGTG) primers for the *gyrB* gene. Sequencing was performed in the Genome sequencing facility as indicated above. The identification tool EzBioCloud was used for pairwise sequence alignment and calculation of similarity scores of the obtained 16S rRNA sequences (Yoon et al., 2017). Based on a list of the 43 best matches in the MEGA X program, a phylogenetic tree was created by using the maximum likelihood method and Kimura two‐parameter model (Kimura, 1980) with 500 bootstrap replications. The obtained *rpoB* and *gyrB* gene sequences were compared and analysed similarly. Sequence similarity was determined by BLAST comparison.

### Genome sequencing

The genomic DNA of strain AB was sequenced using the Illumina MiSeq (Illumina, USA) and Oxford Nanopore Technology (ONT, UK) platforms. The quality of the Illumina MiSeq raw reads was checked using FastQC version 0.11.9 (Andrews et al., 2010), and the reads were subsequently filtered with fastp (Chen et al., 2018). The sequence data obtained from the ONT sequencing data were base‐called with quality filtering (>Q7) using Guppy version 3.6.0. The quality‐filtered sequences of the Illumina MiSeq and ONT reads were assembled using unicycler version 0.4.7 (Wick et al., 2017) to obtain a hybrid genome assembly of strain AB. The quality and completeness of the assembled genome were checked using QUAST 5.0.2 (Gurevich et al., 2013) and PGAP (version 2022‐10‐03.build6384) (Li et al., 2021). rRNA and tRNA screening was performed by running PGAP as described by Li et al. (Li et al., 2021).

### Genome comparison

The genome sequence data were uploaded to the Type (Strain) Genome Server (TYGS), a free bioinformatics platform available at https://tygs.dsmz.de, for whole genome‐based taxonomic analysis (Meier‐Kolthoff & Göker, 2019). The analysis also took advantage of recently introduced methodological updates and features (Meier‐Kolthoff et al., 2022). Information on nomenclature, synonymy, and related taxonomic literature was provided by the sister database of TYGS, the List of Prokaryotic Names with Standing in Nomenclature (LPSN, available at https://lpsn.dsmz.de) (Meier‐Kolthoff et al., 2022). Results were provided by TYGS on 2023‐05‐21 and 2023‐07‐13. The Genome BLAST Distance Phylogeny approach (GBDP) under the algorithm ‘coverage’ and distance formula d5 (Meier‐Kolthoff et al., 2013) and digital DDH (dDDH) values were calculated accordingly (Meier‐Kolthoff et al., 2013; Meier‐Kolthoff et al., 2022). The average nucleotide identity (ANI) values were calculated by the OrthoANIu algorithm using the EzBioCloud identification tool (Yoon et al., 2017).

### Genome analysis

Metabolic pathways were mapped by searching for orthologues in KEGG (Kyoto Encyclopedia of Genes and Genomes) using BlastKOALA (Kanehisa et al., 2016). To search for metabolite biosynthesis gene clusters, antiSMASH 7.0.1 software was used (Blin et al., 2023). The following options were used: KnownClusterBlast, ClusterBlast, SubClusterBlast, MIBiG cluster comparison, ActiveSiteFinder, RREFinder, Cluster Pfam analysis, Pfam‐based GO term annotation, TIGRFam analysis, and TFBS analysis.

The PHASTER web server was used to identify prophage sequences within the AB genome (Arndt et al., 2016). The resistome was predicted with Resistance Gene Identifier (RGI) 5.1.1 using the Comprehensive Antibiotic Resistance Database (CARD) 3.1.1 (Alcock et al., 2019).

## RESULTS

### Phenotyping

The cells of the new strain AB were Gram‐positive, short rods with cell sizes in the range of 0.32–0.56 x 0.76–1.29 μm (mean size 0.42 x 0.97 μm) with an irregular arrangement. They are nonspore‐forming and nonmotile (Figure S1).

The growth and metabolic properties of the new strain are presented in Table 1. Colonies that grew after 48 h of incubation at 28°C on TSA and nutrient agar were grey‐yellowish, matte, small, and slightly convex (Figure S2). These colonies are distinctly different from the colonies of three *Microbacterium* species tested in parallel, which are larger, shiny and flat. The isolate grew within wide temperature and pH ranges (4–45°C and 6.0–12.0, respectively) at a maximum NaCl concentration of 8% (w/v). The optimum growth was observed at 30°C. The strain was aerobic, catalase‐positive, and oxidase‐negative, similar to other *Microbacterium* species examined (Table 1).

**TABLE 1:** Differential characteristics between *Microbacterium betulae* sp. nov. (strain AB) and the type strains of related species of the genus *Microbacterium*.

Phenotypic character	*M. betulae* sp. nov.	*M. barkeri*	*M. sediminis*	*M. ulmi*
Colony morphology^a^	Grey–yellowish, matt, small (1–2 mm), slightly convex	Grey–yellowish, glossy, large (5–7 mm), flat	Grey–yellowish, glossy, mid‐sized (2–3 mm), flat	Grey‐whitish, glossy, large (5–7 mm), flat
Temperature range at which growth occurs	4–45°C	4–55°C	22–45°C	22–45°C
pH range at which growth occurs	6–12	5–12	6–12	7–9
The highest NaCl content at which growth occurs	8%	9%	8%	3%
Catalase	+	+	+	+
Oxidase	−	−	−	−
Indole	−	−	−	−
ENTEROtest results breakdown of:				
Salicin	+	−	−	−
Cellobiose	+	−	−	−
API ZYM results enzymes:				
Esterase lipase (C 8)	+	−	−	−
α‐Glucosidase	+	−	−	−
β‐Glucosidase	+	−	−	−
BIOLOG results utilization of:^b^				
3‐Methyl‐glucose	−	+	+	+
Inosine	−	+	+	+
L‐Alanine	−	+	+	+

^a^ On tryptic soya agar (TSA).

^b^ Utilization as a carbon source.

The metabolic characteristics presented in Table 1 show only the differences between the AB strain and other *Microbacterium* species studied. The AB strain showed higher metabolic activity than the other species studied, e.g., it was positive for the degradation of salicin and cellobiose and contained esterase lipase (C8), α‐glucosidase, and β‐glucosidase.

Regarding the utilization of carbon sources, the AB strain utilized more carbon sources than *M. sediminis* and *M. ulmi* (29 vs. 28 vs. 18, respectively), but only half as much as *M. barkeri* (29 vs. 64 out of a total of 71) (Table S1).

### Chemotaxonomic characterization

Alanine, glycine, glutamic acid, and ornithine were detected in an isolated cell wall of strain AB. Screening for the presence of diaminopimelic acid (DAP) isomers in whole‐cell hydrolysates was negative. Galactose was found among the cell wall sugars.

The predominant fatty acids were anteiso‐branched C15:0 (34.8%) and C17:0 (34.9%), as well as iso‐branched C16:0 (23.3%). The same fatty acid profile was found in *M. barkeri*, *M. sedimini*s, and *M. ulmi* (Table 2). In the AB strain, the smallest value of anteiso C17:0 and the highest value of iso C16:0 were observed among the *Microbacterium* species studied. In addition, differences were found in the lipid profile of the minor fatty acid: a higher value of normal C16:0 than in *M. barkeri* and a lower value of iso C15:0 than in *M. ulmi*. Mycolic acids were not present in the AB strain.

**TABLE 2:** Cellular fatty acids of *M. betulae* sp. nov. (strain AB) and its corresponding reference strains of the *Microbacterium* genus. The values represent percentages of total fatty acids.

Fatty acids	*M. betulae* sp. nov.	*M. barkeri*	*M. sediminis*	*M. ulmi*
iso‐C14:0	0.68	0.15	0.29	0.27
C14:0	0.17	0.02	0.06	0.04
iso‐C15:0	0.53	0.43	0.83	4.32
**anteiso‐C15:0**	**34.81**	**32.72**	**35.49**	**34.22**
C15:0	0.33	0.12	0.13	0.12
**iso‐C16:0**	**23.35**	**16.72**	**21.17**	**12.92**
C16:0	4.73	0.64	2.01	1.65
iso‐C17:0	0.44	0.73	0.53	2.05
**anteiso‐C17:0**	**34.97**	**48.47**	**39.50**	**44.42**

*Note*: Strains: 1, *M. betulae* sp. nov.; 2, *M. barkeri* DSM 20145^T^; 3, *M. sediminis* DSM 23767^T^; 4, *M. ulmi* DSM 16931^T^. All the data were obtained from this study. All strains were cultivated with the same media and growth conditions. The data marked in bold represent the major fatty acids of all strains.

Menaquinone MK‐10 was the major respiratory quinone, with MK‐9, MK‐8 and MK‐11 as minor components (55, 19, 16, and 9%, respectively) (Figure S3). Trace amounts of MK‐12 were also detected (0.2%). The most similar menaquinone profile was reported in *M. sediminis*, with MK‐10 as the major menaquinone (Yu et al., 2013).

The polar lipid profile of the AB strain comprises diphosphatidylglycerol, phosphatidylglycerol, and two unidentified glycolipids (Figure S4). Comparative analysis of the lipid profiles of *M. barkeri*, *M. sediminis*, and *M. ulmi* by 2D‐TLC revealed the same phospholipids, in contrast to the glycolipid profile: one major glycolipid was detected in *M. ulmi* and *M. barkeri* and three in *M. sediminis* (Figure S4).

### Protein profiling

*Microbacterium* species grown in parallel under the same conditions were identified by protein profiling using a MALDI‐TOF MS Biotyper (Table 3). The protein spectra of *M. ulmi* and *M. barkeri* were included in the Biotyper database and these strains were correctly identified, in contrast to those of *M. sediminis* and strain AB, which were not reliably identified (scores below 1.7). In addition, the protein mass spectra generated for the *Microbacterium* strains were distinct. (Figure S5). According to the MALDI dendrogram, the AB isolate formed a cluster that was separated from other *Microbacterium* spp.; to date, *M. sediminis* and *M. ulmi* have shown very similar profiles (Figure S6).

**TABLE 3:** MALDI‐TOF Biotyper identification of *M. betulae* sp. nov. (strain AB), *M. barkeri*, *M. sediminis*, and *M. ulmi* in the Bruker Biotyper database.

Strain	Condition^a^	Organism	Score value^b^
*M. barkeri*	48h_30^o^C_NA_e	*M. barkeri*	2.355
	48h_30^o^C_BL_e	*M. barkeri*	2.331
	48h_30^o^C_TSA_e	*M. barkeri*	2.345
	48h_30^o^C_TSA_d	*M. barkeri*	2.290
*M. betulae* sp. *nov*. (strain AB)	48h_30^o^C_NA_e	NR	1.271
	48h_30^o^C_BL_e	NR	1.434
	48h_30^o^C_TSA_e	NR	1.362
	48h_30^o^C_TSA_d	NR	1.502
*M. sediminis*	48h_30^o^C_NA_e	NR	1.355
	48h_30^o^C_BL_e	NR	1.285
	48h_30^o^C_TSA_e	NR	1.293
	48h_30^o^C_TSA_d	NR	1.301
*M. ulmi*	48h_30^o^C_NA_e	*M. ulmi*	2.166
	48h_30^o^C_BL_e	*M. ulmi*	2.023
	48h_30^o^C_TSA_e	*M. ulmi*	2.242
	48h_30^o^C_TSA_d	*M. ulmi*	2.140

^a^ *Microbacterium* spp. were grown aerobically on tryptic soy agar (TSA), nutrient agar (NA), and blood agar (BL) for 48 h at 30°C. The samples were prepared by the formic acid/acetonitrile extraction method (e) or by the direct transfer method (d).

^b^ Score values determined by MALDI Biotyper: <1.7 – identification not reliable (NR), 1.7–2.0 – probable genus identification, 2.0–2.3 – secure genus identification and probable species identification, > 2.3 – highly probable species identification.

### Phylogenetic analysis

**FIGURE 1:**
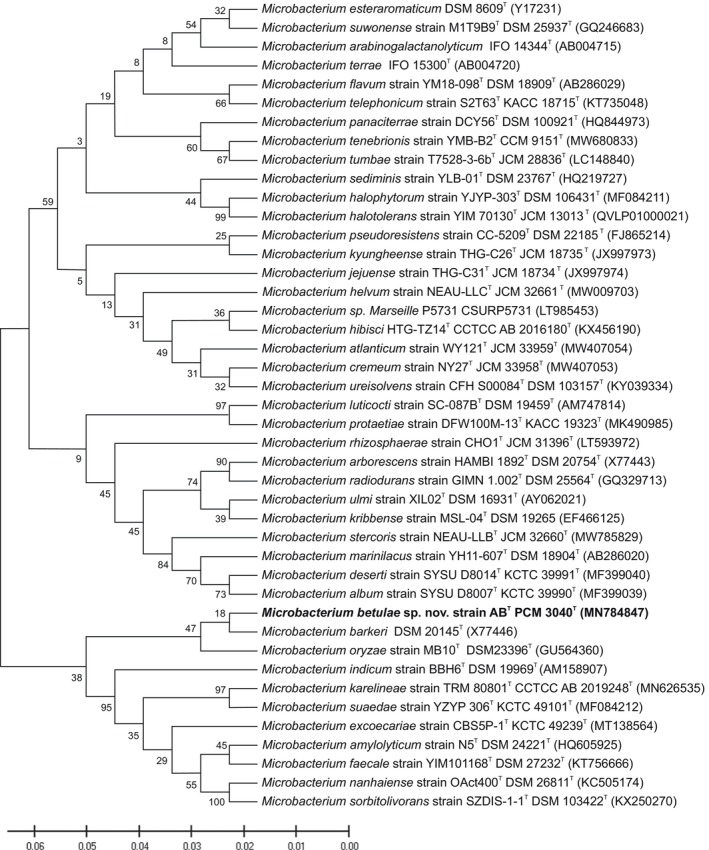
Maximum likelihood phylogenetic tree based on 16S rRNA gene sequences showing the phylogenetic relationships of *M. betulae* sp. nov. to closely related species of the genus *Microbacterium*. Bootstrap values from 500 replications are given at nodes. Bar, 0.01, substitutions per site.

Genomic DNA was isolated from the AB strain, and the 16S rRNA gene was amplified using 16S start and 16S stop primers. The 16S rRNA, *rpoB*, and *gyrB* genes were selected for the initial determination of taxonomic position according to the standards defined by the Clinical and Laboratory Standards Institute (Clinical and Laboratory Standards Institute 2008; Case et al., 2007). Three independent samples were obtained and sequenced using the Sanger method. A comparison of sequences 1353 bp in length in the EZ taxon service showed that the studied strain belongs to the genus *Microbacterium* and is most likely a new species (Figure 1). The maximum likelihood tree constructed based on the 16S rRNA gene sequence of the AB strain and related *Microbacterium* species showed a close relationship to *M. barkeri* and *M. oryzae* (Figure 1).

The 16S rRNA gene (1527 bp) of strain AB showed the highest sequence similarity to that of *M. barkeri* DSM 20145^T^ (Takeuchi et al., 1998; X77446.1), with 97.25%, followed by that of *Microbacterium oryzae* MB10^T^ (Kumari et al., 2013; GU564360.1), with 96.91% similarity, and that of *Microbacterium amylolyticum* DSM 24221^T^ (Anand et al., 2012; HQ605925.1), with 96.27% similarity.

The sequences of the housekeeping genes *rpoB* and *gyrB* were also obtained by PCR and Sanger sequencing. The sequence similarity as determined by BLAST comparison of the two analysed sequences (*rpoB*, and *gyrB*) was as follows: for the *rpoB* gene, *M. oryzae* strain MB10^T^ (CP032550.1) had 93.44% identical bases, *Microbacterium diaminobutyricum* strain RZ102^T^ (KU843546.1) had 90.80%, and *Microbacterium aurum* KACC 15219^T^ (AM181567.1) had 90.67%. Similarity analysis of the *gyrB* gene showed that *M. oryzae* strain MB10^T^ (CP032550.1) had 94.85% of similarity, *Microbacterium flavum* DSM 18909^T^ (KU843509.1) had 86.92%, and *Microbacterium saccharophilum* DSM 28107^T^ (KU843514.1) had 86.85%. Individual phylogenetic trees showing analysis of the *rpoB* and *gyrB* genes of the tested strains and the reference relatives are shown in Figures S7 and S8.

### Genome annotation and analysis

The whole genome was sequenced as described in the Materials and Methods section. The AB strain genome sequence generated 2,502,988 reads from Illumina MiSeq and 11,252 reads from ONT sequencing. A depth coverage of 178× was obtained with a 100% complete genome containing 1 contig of 3636,743 bp. The DNA G + C content was 71.77 mol %. The final genome of strain AB was deposited in the NCBI GenBank under accession number CP118157.

Structural annotation revealed 3363 open reading frames in the genome, of which 3335 potentially encode proteins. The genes encoding rRNA (5S, 16S, 23S) were found to have three copies each, while 45 genes encoding tRNA and three genes encoding ncRNA were identified (Table S2).

**FIGURE 2:**
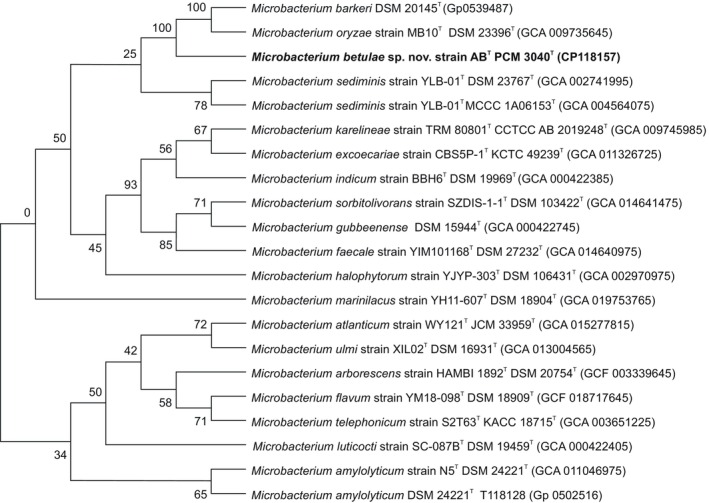
The tree was inferred with FastME 2.1.6.1 (Goloboff et al., 2008) from GBDP distances calculated from genome sequences. The branch lengths are scaled in terms of the GBDP distance formula d5. The numbers above the branches are GBDP pseudobootstrap support values >60% from 100 replications, with an average branch support of 65.4%. The tree was rooted at the midpoint (Farris, 1972).

The AB strain's phylogenetic position was determined by comparing its whole‐genome sequence to the genomes of the type strains used in the 16S RNA gene analysis (Table S3). Similar to the 16S rRNA sequence analysis, strain AB forms a common clade with the species *Microbacterium barkeri* (DSM 20145^T^) and *M. oryzae* (MB10^T^ DSM 23396^T^), which is separated from the other species groups (Figure 2; Table S3).

The pairwise digital DNA–DNA hybridization (dDDH) values between strain AB genome and the selected type strain genomes of *M. barkeri* (DSM 20145^T^), *M. oryzae* (MB10^T^, DSM 23396^T^) and *M. sediminis* (MCCC 1A06153^T^) were 25.5, 24.5, and 21.6%, respectively (Table S4), which are below the standard threshold values (70%) for bacterial species delineation.

The ANI values between strains AB and *M. barkeri* (DSM 20145^T^), *M. oryzae* (MB10^T^ DSM 23396^T^), and *M. ulmi* (XIL02^T^ DSM 16931^T^) were 82.52, 81.57 and 75.96%, respectively (Table S5), which again for all cases were lower than the 95.0% ANI cut‐off value suggested for the bacterial species boundary (Chun & Rainey, 2014).

The annotation of clusters of orthologous genes (COG) assigned 51.7%, 1724 entries of 3335 protein‐coding sequences (CDS) with a specific function (Table S6). Annotation was performed with BlastKOALA software (Kanehisa et al., 2016). The most abundant group was “Carbohydrate metabolism”, covering 243 genes. The other highly abundant groups were ‘Protein families: signalling and cellular processes’ and ‘Environmental information processing’, containing 198 and 182 genes, respectively. Additional groups containing 172 and 163 CDSs were ‘Protein families: genetic information processing’ and ‘Genetic information processing’, respectively. The other groups were characterized by much smaller proportions of assigned genes, such as the ‘Amino Acid Metabolism’ category with 119 genes, the ‘Cellular Processes’ category with 106 genes and the ‘Unclassified Metabolism’ category with 90 genes.

The analysis of secondary metabolite biosynthesis gene clusters using antiSMASH software revealed the presence of six regions with similarity to biosynthetic clusters. The detailed results are summarized in Table S7.

The first region contains 36 ORFs and shows homology to a type III polyketide synthase, and the next region, number 2, contains 25 ORFs and is 100% similar to the beta‐lactone synthesis gene cluster from the *M. oryzae* strain MB10^T^. 60% of the genes in region 3 showed a similarity to lassopeptide synthase from *Kocuria indica* strain DE0236. Region 4 is a type III polyketide synthase. The fifth region is a terpene synthase, similar to the carotenoid biosynthesis‐related gene cluster from *Dietzia* sp. and region 6 is a 9‐ORF siderophore synthase.

A search for phage sequences revealed the presence of one prophage region, 15.7 kB, located in the genome from 813,353 to 829,096 bp. This prophage is incomplete and contains 22 protein‐coding genes, 11 of which are highly similar to phage proteins found in the Virus and Prophage Database, with the remainder showing similarity to bacterial proteins. The viral proteins with the highest homologies are DNA methylase PP_00785 from phage Microb_Min1_NC_009603; integrase PP_00773 from phage Mycoba_Minerva_NC_026584; a hypothetical protein PP_00794 from phage Microb_Min1_NC_009603; gp66 protein PP_00786 from phage PHAGE_Burkho_phi1026b_NC_005284, and putative adenine‐specific methyltransferase PP_00783 from PHAGE_Rhizob_vB_RleM_PPF1_NC_025427. The entire region has an average GC content of 69.22% and is terminated by attL/R integration sites.

The genome sequence was also analysed for the presence of antibiotic‐resistance genes. A search with Abricate did not reveal the presence of any genes associated with drug resistance. Using the Resistance Gene Identifier (RGI) tool, the *vanY* glycopeptide resistance gene was found in the AB genome.

## DISCUSSION

The AB strain was isolated from the birch wood, and despite an environmental origin, it has been demonstrated to induce adverse health effects in sawmill workers as described previously (Mackiewicz et al., 2019). The AB strain was considered a secondary pathogen that is likely to enhance the effects of *P. agglomerans*, which is considered to be the main cause of acute clinical symptoms of hypersensitive pneumonitis (HP), such as ground‐glass opacities and dyspnoea (Mackiewicz et al., 2019). It was demonstrated that the pathogenic reaction is mediated by Th1 cells sensitized by bacterial allergens (Greenberger, 2019; Nogueira et al., 2019). As we demonstrated earlier (Mackiewicz et al., 2019), the main role of *P. agglomerans* and the secondary role of the AB strain in causing clinical pulmonary symptoms were evidenced by the inhalation challenge with strongly diluted cell extracts of both strains. All the patients showed a positive response to *P. agglomerans*, and some of them revealed also a positive response to the AB strain. Positive reactions included a significant decrease in forced vital capacity (FVC), which is characteristic of restrictive impairment of lung function, rales in the auscultation examination, increased interleukin 6 (IL‐6) blood levels, and subjective symptoms (cough mainly) (Mackiewicz et al., 2019).

To date, microbacteria are rarely identified as pathogens in human clinical specimens, e.g., a reference centre collected only 50 isolates over 5 years (Gneiding et al., 2008); another study showed that *Microbacterium yannicii* could be considered as an opportunistic human pathogen, especially in immunocompromised patients (Sharma et al., 2012). Therefore, it is plausible to consider the AB strain as potentially pathogenic to humans.

Here the AB strain has been classified as a new species and given the name *Microbacterium betulae* sp. nov. based on a comprehensive phenotypic, chemotaxonomic, phylogenetic and whole‐genome analysis.

The *M. betulae* AB strain exhibited significant levels of enzyme and metabolic activity (Table 1). These results are even more intriguing in light of the strain's wood origin, e.g., salicin is an alcoholic β‐glucoside that is produced in the bark and leaves of trees, mostly willows (*Salix*) and poplars, while cellobiose is a disaccharide obtained by hydrolysis of cellulose, thus, it is possible that these enzymes appeared in wood‐inhabiting *M. betulae* as a result of evolutionary adaptation. Examples of such adaptations have been reported in a number of environments (Anderson et al., 2021; Ulusu, 2015). Likewise, the wood origin of these bacteria may also be connected to the presence of certain enzymes found by API ZYM tests: Godse et al. (2021) identified α‐glucosidase as a key enzyme responsible for the breakdown of lignocellulosic biomass, and its presence is also compatible with the degradation of salicin (Anderson et al., 2021; Ulusu, 2015). Esterase lipase (C8) was detected in our isolate, which is consistent with esterases' well‐known ability to degrade plant cell walls (Biely, 2012). To note, *M. betulae* and *M. ulmi* both produce esterase (C4), another enzyme from that group (Table 4). Notably, the latter bacteria were also isolated from an elm tree (*Ulmus nigra*) in Spain (Rivas et al., 2004). In general, the novel AB strain's adaptability to wood colonization was confirmed by its high enzymatic activity and resilience to adverse environmental conditions.

**TABLE 4:** Protologue description of *Microbacterium betulae* sp. nov.

Guiding code for nomenclature	ICNP
Nature of the type of material	Strain and genome sequence
Genus name	*Microbacterium*
Species name	*Microbacterium betulae*
Specific epithet	*betulae*
Species status	sp. nov.
Species etymology	*Microbacterium betulae* sp. nov. (be.tu'lae L. fem. n. betula birch; N.L. gen. fem. *betulae* of birch, referring to the source of isolation)
Designation of the Type Strain	AB^T^
Strain Collection Numbers	PCM 3040^T^ = CECT 30706^T^
Type Genome, MAG or SAG accession Nr. [INSDC databases]	CP118157^TS^
Genome status	Complete
Genome size	3636
GC mol%	71.77
16S rRNA gene accession nr.	MN784847.1
Description of the new taxon and diagnostic traits	Cells are Gram‐stain‐positive, aerobic, non‐spore‐forming, and nonmotile short rods (0.76–1.29 μm long and 0.32–0.56 μm wide) with an irregular arrangement. Colonies are grey–yellowish, matt, small, circular, and slightly convex. The cultures grow well on TSA agar, nutrient agar, BHI agar, and nutrient agar supplemented with 5% sheep blood (without haemolysis). The isolate grew within the wide temperature and pH ranges (4–45°C) and 6.0–12.0, respectively, at a maximum NaCl concentration of 8%. The strain is aerobic, catalase‐positive, and oxidase‐negative. API ZYM kit revealed positive reactions for esterase (C4), esterase lipase (C8), leucine arylamidase, naphtol‐AS‐B1‐phosphohydrolase, α‐glucosidase, and β‐glucosidase. Negative tests were obtained for alkaline phosphatase, lipase (C14), valine arylamidase, cystine arylamidase, trypsin, α‐chymotrypsin, acid phosphatase, α‐galactosidase, β‐ galactosidase, β‐glucuronidase, N‐acetyl‐β‐glucosaminidase, α‐mannosidase, and α‐fucosidase. Carbon source utilization ability determined by Biolog GEN III Microplate test panel was positive for dextrin, D‐maltose, D‐trehalose, D‐cellobiose, gentiobiose, sucrose, D‐turanose, stachyose, D‐raffinose, D‐melibiose, D‐salicin, α‐D‐glucose, D‐fructose, D‐galactose, D‐mannitol, myoinositol, pectin, D‐gluconic acid, L‐lactic acid, and acetoacetic acid. Utilization test results were negative for α‐D‐lactose, N‐acetyl‐β‐D‐mannosamine, N‐acetyl‐D‐galactosamine, N‐acetyl neuraminic acid, 3‐methyl glucose, D‐ and L‐fucose, inosine, D‐sorbitol, D‐glucose 6‐phosphate, D‐aspartic acid, D‐ and L‐serine, gelatin, glycyl‐L‐proline, L‐alanine, L‐arginine, L‐aspartic acid, L‐glutamic acid, L‐histidine, L‐pyroglutamic acid, D‐galacturonic acid, L‐galactonic acid lactone, D‐glucuronic acid, mucic acid, quinic acid, D‐saccharic acid, p‐hydroxyphenyl acetic acid, methyl pyruvate, D‐lactic acid methyl ester, α‐ketoglutaric acid, D‐ and L‐malic acid, bromo‐succinic acid, γ‐amino‐butyric acid, α‐hydroxy‐butyric acid, β‐hydroxy‐D, L‐butyric acid, α‐keto‐butyric acid, propionic acid, acetic acid and formic acid. In chemical sensitivity assays, cells are sensitive towards fusidic acid, D‐serine, troleandomycin, rifamycin RV, minocycline, lincomycin, guanine hydrochloride, Niaproof 4, vancomycin, tetrazolium violet, tetrazolium blue, lithium chloride, and sodium bromate, while cells are resistant to 1% sodium lactate, nalidixic acid, potassium tellurite, aztreonam, and sodium butyrate. The cell‐wall peptidoglycan contains glycine, alanine, glutamic acid, and ornithine. The polar lipids contain diphosphatidylglycerol, phosphatidylglycerol, and unidentified glycolipids. The major fatty acids are anteiso C15:0, anteiso C17:0, and iso C16:0. MK‐10 is the predominant respiratory quinone.
Country of origin	Poland
Region of origin	Lublin province
Date of isolation (dd/mm/yyyy)	17/10/2017
Source of isolation	Pulverized birch wood
Sampling date (dd/mm/yyyy)	03/10/2017
Latitude (xx°xx'xx″N/S)	50°22′18.944”N
Longitude (xx°xx'xx″E/W)	23°27′17.507″ E
Altitude (metres above sea level)	257–327.6 m above sea level (Bełżec)
Number of strains in the study	1
Information related to the Nagoya Protocol	The strain was isolated in Poland and is not subject to regulations. (not applicable)

Chemotaxonomic characteristics, such as the primary menaquinone, the type of murein, and the composition of the cell wall sugars, can be used to differentiate between different species of *Microbacterium* spp. Although it can be difficult to identify *Microbacterium* only based on such characteristics, new isolates of the bacterium can be recognized at the species level by combining chemotaxonomic features with physiological and growth parameters. The chemotaxonomic features of the AB strain are similar to those of the *Microbacterium* genus, such as type B peptidoglycan (the cell wall contains either ornithine or lysine).

The composition of cell wall sugars, such as glucose, galactose, rhamnose, fucose, 6‐deoxytalose, and xylose, varies between strains and species of *Microbacterium*; galactose was the predominant cell wall sugar in the AB strain, but rhamnose was observed in *M. barkeri*. Ribose and glucose were found in *M. sediminis* (Yu et al., 2013), and xylose and fucose were found in *M. ulmi* (Rivas et al., 2004), respectively, in addition to galactose. Galactose was the only carbohydrate found so far in *M. keratanolyticum* and *M. arabinogalactanolyticum* (Evtushenko & Takeuchi, 2006).

The presence of branched iso‐ and anteiso‐fatty acids, the most common of which are anteiso‐C15:0, iso‐C16:0, and anteiso‐C17:0, and the absence of mycolic acids identify the *Microbacterium* taxon. As shown in Table 2, there were no significant differences in the fatty acid profile of *M. barkeri*, *M. sediminis*, and *M. ulmi* when grown in the same medium and at the same growth stage.

*Microbacterium* spp. are dominated by long, unsaturated menaquinones with 10, 11, and 12 isoprene units (Evtushenko & Takeuchi, 2006). In contrast, MK‐13 and MK‐14 are dominant in *M. sulfonylureivorans* and *M. flavescens*, while MK‐8, MK‐9, and MK‐10 are present in *M. sediminis* (Yu et al., 2013).

*M. aurum*, *M. dextranolyticum*, *M. lacticum*, and *M. laevaniformans* were found to contain the major polar lipids, diphosphatidylglycerol, phosphatidylglycerol, and the glycolipid dimannosyl‐diacylglycerol but may also contain traces of monomannosyldiacylglycerol, phosphoglycolipid, and unknown glycolipids (Evtushenko & Takeuchi, 2006). While *M. ulmi* and *M. barkeri* had only one glycolipid, *M. betulae* contained two unidentified glycolipids, and *M. sediminis* was found to have three glycolipids. This feature can therefore be used to distinguish between closely related species of *Microbacterium*.

The AB strain and the other microbacteria examined have different protein profiles, according to the MALDI‐TOF analysis. Furthermore, the MALDI‐TOF MS Biotyper identification results (Table 3) show that they are not strongly influenced by the type of medium. There are 70 *Microbacterium* species in the Bruker Biotyper database (DB 8469), which we used for our investigation, including one *M. barkeri* species and one *M. ulmi* species. Consequently, the unique protein mass spectrum of the AB strain and the low score value (below 1.5) obtained in the MALDI‐TOF study imply that this strain might be a member of the novel species.

16S rRNA analysis and housekeeping gene analysis (*gyrB* and *rpoB*) confirmed that the AB strain belongs to the genus *Microbacterium* and is distinct from known *Microbacterium* species. The genome of *M. betulae* falls within the range of closely related microbacterial genomes. The smallest genome was *M. amylolyticum* with a size of 2,588,343 bp, while the largest was *M. karelineae* with a size of 3,950,561 bp (Table S3). The DNA G + C content of the new strain AB was 71.77 mol%, which falls within the range of 69–75 mol% for the genus *Microbacterium* (Evtushenko & Takeuchi, 2006).

ANI and DDH (DNA–DNA hybridization) have been demonstrated to be useful indices in species delineation. Digital DDH estimation of the strain AB against the compared genomes ranged between 25.5 and 19.1 (Table S4). These values are very low and below the cut‐off of 70%, thus confirming again the new species status of the strain AB. Also, the ANI analysis of the AB strain against the compared genomes ranged between 82.52 and 75.06 (Table S5) and was below the 95.0% ANI cut‐off value.

Analysis of the functional classification of COG genes revealed that the AB strain can recognize a large number of genes, including those involved in metabolism, information processing and storage, and cell processing signalling, for example. The high proportion of regulatory genes and genes related to the reception of environmental signals demonstrates that *M. betulae* is not a typical pathogen (i.e., that depends on host infection for its survival) (Mo et al., 2022) but possesses a high capacity to survive under different environmental conditions.

The adaptability of *M. betulae* to a variety of environmental conditions was confirmed by genomic analysis. Secondary metabolite gene cluster analysis of *M. betulae* sp. nov. revealed terpenoids, type III polyketide synthase (PKS), and siderophore (Table S7). According to studies performed by Corretto et al., who had analysed 70 genomes of *Microbacterium*, the most common in microbacteria were gene clusters for the production of terpenoids and type III polyketide synthase, present in 96% and 79% of the analysed genomes, respectively (Corretto et al., 2020). There have also been reports of C50 carotenoids being produced by *M. luteum*, *M. cremeum*, and *M. atlanticum* (Xie, Niu, et al., 2021).

A sequence of 15.7 kB in length, a probable prophage, was also found in the *M. betulae* genome. According to Jacobs‐Sera et al. (2020), the size range of typical phages in the genus *Microbacterium* is 17.3–97.7 kB; however, the size of this prophage is less. The G + C% content of 69.22% fits in the upper part of the range (51.4 to 71.4) but is lower than the host GC content of 71.77%. The phage contains 22 open reading frames of which only 11 have high similarity to phage proteins (Jacobs‐Sera et al., 2020). To date, it has an integrase with high homology to a *Mycobacterium* phage Minerva, other phage‐specific proteins were not detected. Whether this prophage sequence is active, or it is just a remnant phage (other fragments may have been lost during evolution), is currently difficult to determine.

The genome of *M. betulae* contains the *vanY* glycopeptide resistance gene, which suggests that the organism may be resistant to glycopeptide antibiotics. Genes of this type are frequently linked to processes that modify or degrade glycopeptide antibiotics, allowing bacteria to withstand the therapy. Notably, the final effect of resistance is dependent on the environmental conditions, factors related to the translation or other regulatory factors.

To note, the scope of this study has been restricted to a single strain of *M. betulae* that belongs to a newly discovered species. Research in the same ecological niche has to be conducted to identify further strains, which will be necessary to corroborate its properties.

The strain's interactions with other microbes, particularly fungi, that live in birch wood are another factor. In exploring the bacterial and fungal community of grapevine wood, Haidar et al. (Haidar et al., 2021) suggested that some strains may synergistically interact to enhance or decrease the degradation of grapevine wood, additionally, a strain of *Microbacterium* sp. revealed properties that inhibited the growth of mycelial growth in *Fomitiporia mediterranea* through the secretion of volatile compounds.

The in vitro cultures of a new AB strain in the presence of fungal strains isolated from the same source (e.g., *Penicillium expansum*, *P. glabrum*, *Mucor* spp., *Trichoderma* spp., *Paecilomyces* spp.) (Mackiewicz et al., 2019) may provide important insights into interactions related to microbial competition, symbiosis or effects on wood decay processes within the microbial community. Further investigation of *M. betulae* colonization of wood from different tree species will shed light on the metabolic processes of these bacteria and their general function within the complex wood microbiome.

## CONCLUSIONS

The AB strain, isolated from birch wood, has been identified as a new species within the *Microbacterium* genus, based on comprehensive analysis including 16S rRNA, whole genome sequencing, in silico DNA–DNA hybridization, phenotyping, chemotaxonomy, and MALDI‐TOF MS profiling. This new species has been proposed to be named *M. betulae* sp. nov. The type strain, AB^T^ (PCM 3040 ^T^ = CEST 30706 ^T^), was derived from pulverized birch wood collected in the Lublin province of Poland. The genomic DNA of the type strain has a G + C content of 71.77 mol%. A detailed description of the strain is provided in the Protologue table (Table 4). For comparative studies, strain AB^T^ has been deposited in two culture collections, PCM 3040 ^T^ and CEST 30706 ^T^.

## AUTHOR CONTRIBUTIONS

**Mariola Paściak:** Conceptualization (equal); data curation (equal); formal analysis (equal); funding acquisition (lead); project administration (lead); supervision (equal); visualization (equal); writing – original draft (equal); writing – review and editing (equal). **Krzysztof J. Pawlik:** Data curation (equal); formal analysis (equal); methodology (equal); software (equal); visualization (equal); writing – original draft (equal); writing – review and editing (equal). **Dariusz Martynowski:** Data curation (equal); formal analysis (equal). **Łukasz Łaczmański:** Data curation (equal); formal analysis (equal); methodology (equal); software (equal). **Jarosław Ciekot:** Formal analysis (equal). **Bogumiła Szponar:** Resources (equal); writing – review and editing (equal). **Angelina Wójcik‐Fatla:** Investigation (equal); resources (equal); writing – review and editing (equal). **Barbara Mackiewicz:** Conceptualization (equal); investigation (equal). **Ewelina Farian:** Investigation (equal); writing – review and editing (equal). **Grażyna Cholewa:** Investigation (equal). **Alicja Cholewa:** Investigation (equal). **Jacek Dutkiewicz:** Conceptualization (equal); resources (equal); supervision (equal); writing – original draft (equal); writing – review and editing (equal).

## CONFLICT OF INTEREST STATEMENT

The authors declare no conflicts of interest.

## Supporting information


**FIGURE S1.** Scanning electron micrographs of *M. betulae* sp. nov. after growth on TSA agar at 28°C for 48 h. Bar, 1.0 μm.
**FIGURE S2.** Colony morphology of *M. betulae* sp. nov. after growth on nutrient agar at 28°C for 48 h.
**FIGURE S3.** HPLC analysis of *M. betulae* sp. nov. showing the compositions of menaquinones.
**FIGURE S4.** Two‐dimensional thin layer chromatograms (2D‐TLC) of polar lipids from *M. betulae* sp. nov., *M. barkeri*, *M. sediminis*, and *M. ulmi*. Solvent systems: chloroform–methanol–water (65:25:4, v/v/v) was used in the first dimension (1), and chloroform–methanol–acetic acid–water (80:15:12:4, v/v/v/v) was used in the second dimension (2). Phospholipids (panel A) were detected using the Dittmer & Lester reagent, and total lipids (panel B) were detected using the phosphomolybdic acid reagent. Abbreviations: DPG, diphosphatidylglycerol; PG, phosphatidylglycerol; GL, glycolipid.
**FIGURE S5.** MALDI‐TOF protein mass spectra of *M. barkeri* (A), *M. betulae* sp. nov. (B), *M. sediminis* (C), and *M. ulmi* (D).
**FIGURE S6.** Cluster analysis of protein mass spectra of *M. sediminis*, *M. ulmi*, *M. barkeri*, and *M. betulae* sp. nov. generated in MALDI Biotyper 3.0.
**FIGURE S7.** Maximum likelihood phylogenetic tree based on *rpoG* gene sequences showing the phylogenetic relationships of *M. betulae* sp. nov. to the closely related species of the genus *Microbacterium*. Bootstrap values from 500 replications are given at nodes. GenBank accession numbers and ranges are given before the organism's name.
**FIGURE S8.** Maximum likelihood phylogenetic tree based on *gyrB* gene sequences showing the phylogenetic relationships of *M. betulae* sp. nov. to the closely related species of the genus *Microbacterium*. Bootstrap values from 500 replications are given at nodes. GenBank accession numbers and ranges are given before the organism's name.
**TABLE S1.** Utilization of carbon sources by *M. betulae* sp. nov. strain AB and the type strains of closely related *Microbacterium* species. All data in the table are from the present study.
**TABLE S2.** Assembly and structural annotation of the genome of *Microbacterium betulae* sp. nov. PCM 3040^T^ strain AB^T^.
**TABLE S3.** List of type strains *Microbacterium* genomes closely related to *M. betulae* sp. nov. strain AB created in TYGS.
**TABLE S4.** Pairwise comparisons of *M. betulae* sp. nov. strain AB genome vs. type‐strain genomes of closely related *Microbacterium* species. The table contains the pairwise dDDH values between the *M. betulae* AB strain genome and the selected type strain genomes. The dDDH values are provided along with their confidence intervals (C.I.) for the three different GBDP formulas: formula d0 (GGDC formula 1): length of all HSPs divided by total genome length; formula d4 (GGDC formula 2): sum of all identities found in HSPs divided by overall HSP length; formula d6 (GGDC formula 3): sum of all identities found in HSPs divided by total genome length.
**TABLE S5.** Ortho ANI values for *M. betulae* sp. nov. strain AB and the type strains of closely related *Microbacterium* species.
**TABLE S6.** Clusters of orthologous groups (COG) of the *Microbacterium. betulae* sp. nov. PCM 3040^T^ strain AB^T^.
**TABLE S7.** Secondary metabolite regions identified within the genome sequence of the *M. betulae* sp. nov. PCM 3040^T^ strain AB^T^ using the antiSMASH version 7.0 algorithm.

## Data Availability

The partial sequence of the 16S ribosomal RNA gene from the *Microbacterium barkeri* strain PCM 3040 has been deposited in GenBank under the accession number MN784847.1. Additionally, the complete genome of the *Microbacterium* sp. AB strain, specifically the AB chromosome, is also available in GenBank under the accession number CP118157.1.
