# Supplementary material for: Discovery of a new bacterium, Microbacterium betulae sp. nov., in birch wood associated with hypersensitivity pneumonitis in woodworkers
Source: Environ Microbiol Rep. 2024 Aug 12;16(4):e13311. doi: 10.1111/1758-2229.13311 (PMC11319209; doi:10.1111/1758-2229.13311)
Supplement: Supplementary file 1 — FIGURE S1. Scanning electron micrographs of M. betulae sp. nov. after growth on TSA agar at 28°C for 48 h. Bar, 1.0 μm. FIGURE S2. Colony morphology of M. betulae sp. nov. after growth on nutrient agar at 28°C for 48 h. FIGURE S3. HPLC analysis of M. betulae sp. nov. showing the compositions of menaquinones. FIGURE S4. Two‐dimensional thin layer chromatograms (2D‐TLC) of polar lipids from M. betulae sp. nov., M. barkeri, M. sediminis, and M. ulmi. Solvent systems: chloroform–methanol–water (65:25:4, v/v/v) was used in the first dimension (1), and chloroform–methanol–acetic acid–water (80:15:12:4, v/v/v/v) was used in the second dimension (2). Phospholipids (panel A) were detected using the Dittmer & Lester reagent, and total lipids (panel B) were detected using the phosphomolybdic acid reagent. Abbreviations: DPG, diphosphatidylglycerol; PG, phosphatidylglycerol; GL, glycolipid. FIGURE S5. MALDI‐TOF protein mass spectra of M. barkeri (A), M. betulae sp. nov. (B), M. sediminis (C), and M. ulmi (D). FIGURE S6. Cluster analysis of protein mass spectra of M. sediminis, M. ulmi, M. barkeri, and M. betulae sp. nov. generated in MALDI Biotyper 3.0. FIGURE S7. Maximum likelihood phylogenetic tree based on rpoG gene sequences showing the phylogenetic relationships of M. betulae sp. nov. to the closely related species of the genus Microbacterium. Bootstrap values from 500 replications are given at nodes. GenBank accession numbers and ranges are given before the organism's name. FIGURE S8. Maximum likelihood phylogenetic tree based on gyrB gene sequences showing the phylogenetic relationships of M. betulae sp. nov. to the closely related species of the genus Microbacterium. Bootstrap values from 500 replications are given at nodes. GenBank accession numbers and ranges are given before the organism's name. TABLE S1. Utilization of carbon sources by M. betulae sp. nov. strain AB and the type strains of closely related Microbacterium species. All data in the table are from the pr [file EMI4-16-e13311-s001.pdf]

Supporting Information

Table S1. Utilization of carbon sources by *M. betulae* sp. nov. strain AB and the type strains of closely related *Microbacterium* species. All data in the table are from the present study.

Carbon source	AB strain	*M. barkeri*	*M. sediminis*	*M. ulmi*
Dextrin	+	+	+	+
D-Maltose	+	+	+	(-)
D-Trehalose	+	+	+	+
D-Cellobiose	+	+	+	+
Gentiobiose	+	+	(-)	(-)
Sucrose	+	+	(-)	(-)
D-Turanose	+	+	±	(-)
Stachyose	+	+	(-)	(-)
D-Raffinose	+	+	(-)	(-)
α-D-Lactose	(-)	+	(-)	(-)
D-Mellibiose	+	+	(-)	(-)
β-Methyl-D Glucoside	+	+	(-)	(-)
D-Salicin	+	+	+	(-)
N-Acetyl-D Glucosamine	±	+	(-)	(-)
N-Acetyl-D Mannosamine	(-)	±	(-)	(-)
N-Acetyl-D Galactosamine	(-)	(-)	(-)	(-)
N-Acetyl Neuraminic Acid	(-)	(-)	(-)	(-)
α-D-Glucose	+	+	+	+
D-Mannose	±	+	+	+
D-Fructose	+	+	+	+
D-Galactose	+	+	+	(-)
D-Fucose	(-)	±	±	(-)
L-Fucose	(-)	±	(-)	(-)
Inosine	(-)	+	±	+

Carbon source	AB strain	*M. barkeri*	*M. sediminis*	*M. ulmi*
D-Sorbitol	(-)	(-)	(-)	(-)
D-Mannitol	+	+	+	+
D-Arabitol	±	+	(-)	(-)
myo-Inositol	+	±	(-)	(-)
Glycerol	±	+	(-)	(-)
D-Glucose-6-PO4	(-)	±	±	(-)
D-Fructose-6-PO4	(-)	±	±	(-)
D-Aspartic Acid	(-)	±	(-)	(-)
D-Serine	(-)	(-)	(-)	(-)
Gelatin	(-)	+	(-)	(-)
Glycyl-L-Proline	(-)	+	±	(-)
L-Alanine	(-)	+	±	+
L-Arginine	(-)	±	(-)	(-)
L-Glutamic Acid	(-)	+	(-)	(-)
L-Histidine	(-)	+	(-)	(-)
L-Pyroglutamic Acid	(-)	±	±	(-)
L-Serine	(-)	±	(-)	(-)
Pectin	+	+	(-)	(-)
D-Galacturonic Acid	(-)	+	±	(-)
L-Galactonic Acid Lactone	(-)	±	±	(-)
D-Gluconic Acid	+	+	+	+
L-Rhamnose	±	+	(-)	+
Mucic Acid	(-)	±	(-)	(-)
D-Saccharic Acid	(-)	±	(-)	(-)

Carbon source	AB strain	*M. barkeri*	*M. sediminis*	*M. ulmi*
p-Hydroxy Phenylactic Acid	(-)	+	(-)	(-)
Methyl Pyruvate	(-)	+	(-)	+
D-Lactic Acid Methyl Ester	(-)	±	(-)	(-)
L-Lactic Acid	+	+	±	+
Citric Acid	±	+	(-)	(-)
α-Keto-Glutaric Acid	(-)	+	(-)	(-)
D-Malic Acid	(-)	+	(-)	(-)
L-Malic Acid	(-)	+	+	(-)
Bromo-Succinic Acid	(-)	+	(-)	(-)
Tween 40	±	+	±	+
γ-Amino-Butyric Acid	(-)	(-)	(-)	(-)
α-Hydroxy Butyric Acid	(-)	±	(-)	(-)
β-Hydroxy-D,L-ButyricAcid	(-)	(-)	(-)	(-)
α-Keto-Butyric Acid	(-)	±	(-)	(-)
Acetoacetic Acid	+	+	+	+
Propionic Acid	(-)	±	(-)	(-)
Acetic Acid	(-)	+	±	(-)
Formic Acid	(-)	(-)	(-)	(-)
L-Aspartic Acid	(-)	+	(-)	(-)
3-Methyl-Glucose	(-)	±	±	+
D-Glucuronic Acid	(-)	±	(-)	±
Glucuronamide	±	±	±	±
Quinic Acid	(-)	±	(-)	(-)

Legend: + (positive); ± (borderline positive); (-) (negative)

Table S2. Assembly and structural annotation of the genome of *Microbacterium betulae* sp. nov. PCM 3040^T strain AB^T.

**Feature**	
Length (bp)	3 636 743 bp
No. of contigs	1
Genome coverage	130.0x
GC content (mol%)	71.77
Total no. of ORFs	3 363
Protein coding genes	3 335
Pseudogenes	28
rRNA genes (5S, 16S, 23S)	3, 3, 3
tRNA	45
ncRNA	3

Table S3. List of type strains *Microbacterium* genomes closely related to *M. betulae* sp. nov. strain AB created in TYGS

TYGS ID	Kind	Species cluster	Subspecies cluster	Preferred name	Deposit	Other deposits	Base pairs	Percent G+C	No. proteins	Goldstamp	Bioproject accession	Biosample accession	Assembly accession
11663	type strain	1	0	Microbacterium telephonicum	S2T63	MCC 2967; LMG 29293; KACC 18715	3 112 560	70.96	2897	Gp0248681	PRJNA440731	SAMN08775494	GCA_003651225
118128	type strain	2	6	Microbacterium amylolyticum	DSM 24221	CCM 7881; N5	2 588 343	64.52	2498	Gp0502516			
13969	type strain	2	6	Microbacterium amylolyticum	DSM 24221	CCM 7881; N5	2 588 344	64.52	2469		PRJNA608228	SAMN14167854	GCA_011046975
118136	type strain	3	1	Microbacterium barkeri	DSM 20145	NCIMB 9658; ATCC 15954; CCM 1928; IMET 10688; NCIB 9658	3 511 699	71.43	3246	Gp0539487			
12086	type strain	4	2	Microbacterium halophytorum	YJYP303	CGMCC 1.16264; KCTC 49100	3 326 061	70.77	2919	Gp0376011	PRJNA427966	SAMN08278568	GCA_002970975
121549	type strain	5	3	Microbacterium flavum	DSM 18909	JCM 15574; MBIC 8278; YM 18-098	3 260 063	70.35	3030		PRJNA224116	SAMN18119817	GCF_018717645
125838	type strain	6	4	Microbacterium ulmi	JCM 14282	LMG 20991; CECT 5976; DSM 16931; JCM 14282; XIL02	3 495 776	70.77	3233		PRJNA622446	SAMN14517836	GCA_013004565
131970	type strain	7	5	Microbacterium excoecariae	CBS5P-1	CGMCC 1.13862; KCTC 49239	2 710 784	71.84	2442		PRJNA610138	SAMN14278075	GCA_011326725
1419	type strain	8	7	Microbacterium indicum	DSM 19969	LMG 23459; JCM 21800; BBH6; IAM 15355	2 811 946	71.42	2644	Gp0013052	PRJNA188869	SAMN02440686	GCA_000422385
1427	type strain	9	8	Microbacterium luticocti	DSM 19459	CCUG 54537; JCM 15576; SC-087B	3 113 993	70.73	2828	Gp0013053	PRJNA188870	SAMN02440801	GCA_000422405
157862	type strain	10	9	Microbacterium sediminis	MCCC 1A06153	MCCC 1A06153; DSM 23767; CCTCC AB 2010363; ylb-01	2 919 864	71.62	2709		PRJNA528380	SAMN11180679	GCA_004564075
158049	type strain	10	9	Microbacterium sediminis	YLB-01	MCCC 1A06153; DSM 23767; CCTCC AB 2010363; ylb-01	2 755 803	71.9	2599	Gp0124577	PRJNA261778	SAMN03075694	GCA_002741995
158244	type strain	11	10	Microbacterium marinilacus	YM11-607	DSM 18904; JCM 16546; MBIC 7778	3 886 725	71.7	3626		PRJNA755494	SAMN20826684	GCA_019753765
1611	type strain	12	11	Microbacterium gubbeenense	DSM 15944	LMG S-19263; NCIMB 30129; DSM 24532; JCM 12075; NBRC 103073; DPC 5286	3 017 774	68.14	2864	Gp0013051	PRJNA185602	SAMN02440649	GCA_000422745
40936	type strain	13	12	Microbacterium faecale	CGMCC 1.15152	KCTC 39554; DSM 27232; YIM 101168	3 115 067	68.12	2968		PRJDB10509	SAMD00245022	GCA_014640975
48424	type strain	14	13	Microbacterium sorbitolivorans	CGMCC 1.15228	CGMCC 1.15228; DSM 103422; C1.15228; SZDIS-1-1	3 049 715	68.49	2833		PRJDB10509	SAMD00245028	GCA_014641475
6522	type strain	15	14	Microbacterium arborescens	DSM 20754	LMG 4009; CIP 55.81; ATCC 4358; JCM 5884; IFO 3750; NBRC 3750; VKM Ac-1944; HAMBI 1892	3 390 579	69.32	3162	Gp0393597	PRJNA224116	SAMN09428468	GCF_003339645
80983	type strain	16	15	Microbacterium karelineae	TRM 80801	KCTC 49357; CCTCC AB 2019248	3 950 561	70.07	3581		PRJNA577614	SAMN13030836	GCA_009745985
81254	type strain	17	16	Microbacterium atlanticum	WY121	MCCC 1A16761; JCM 33959	3 712 988	70.72	3347		PRJNA673753	SAMN16453562	GCA_015277815
8325	type strain	18	17	Microbacterium oryzae	MB10	DSM 23396; JCM 16837	3 040 780	71.1	2804		PRJNA224116	SAMN10053049	GCA_009735645
U437434	user strain	19	18	AB.contigs			3 581 842	71.35	5003				

Table S4 Pairwise comparisons of *M. betulae* sp. nov. strain AB genome vs. type-strain genomes of closely related *Microbacterium* species.

The table contains the pairwise dDDH values between *M. betulae* AB strain genome and the selected type strain genomes. The dDDH values are provided along with their confidence intervals (C.I.) for the three different GBDP formulas: formula d0 (GGDC formula 1): length of all HSPs divided by total genome length; formula d4 (GGDC formula 2): sum of all identities found in HSPs divided by overall HSP length; formula d6 (GGDC formula 3): sum of all identities found in HSPs divided by total genome length.

**Query strain**	**Subject strain**	**dDDH (d0, in %)**	**C.I. (d0, in %)**	**dDDH (d4, in %)**	**C.I. (d4, in %)**	**dDDH (d6, in %)**	**C.I. (d6, in %)**	**G+C content difference (in %)**
'AB.contigs'	Microbacterium barkeri DSM 20145	31.01	[27.8 - 34.7]	25.5	[23.2 - 28.0]	28.8	[25.9 - 31.9]	0.09
'AB.contigs'	Microbacterium oryzae MB10	29.7	[26.3 - 33.3]	24.5	[22.2 - 27.0]	27.4	[24.5 - 30.5]	0.25
'AB.contigs'	Microbacterium sediminis MCCC 1A06153	20.7	[17.5 - 24.4]	21.6	[19.4 - 24.1]	20.0	[17.3 - 23.0]	0.27
'AB.contigs'	Microbacterium sediminis YLB-01	20.8	[17.6 - 24.4]	21.5	[19.2 - 23.9]	20.0	[17.3 - 23.1]	0.56
'AB.contigs'	Microbacterium marinilacus YM11-607	20.4	[17.2 - 24.0]	21.2	[18.9 - 23.6]	19.6	[16.9 - 22.7]	0.35
'AB.contigs'	Microbacterium halophytorum YJYP303	17.6	[14.5 - 21.1]	21.0	[18.8 - 23.5]	17.3	[14.7 - 20.3]	0.58
'AB.contigs'	Microbacterium karelineae TRM 80801	18.9	[15.8 - 22.5]	20.8	[18.6 - 23.2]	18.4	[15.7 - 21.4]	1.28
'AB.contigs'	Microbacterium excoecariae CBS5P-1	20.1	[17.0 - 23.7]	20.5	[18.2 - 22.9]	19.4	[16.7 - 22.4]	0.49
'AB.contigs'	Microbacterium indicum DSM 19969	21.9	[18.7 - 25.5]	20.5	[18.2 - 22.9]	20.7	[18.0 - 23.8]	0.08
'AB.contigs'	Microbacterium ulmi JCM 14282	17.8	[14.7 - 21.3]	20.5	[18.3 - 22.9]	17.4	[14.8 - 20.4]	0.58
'AB.contigs'	Microbacterium sorbitolivorans CGMCC 1.15228	17.8	[14.7 - 21.3]	20.3	[18.1 - 22.7]	17.4	[14.8 - 20.4]	2.86
'AB.contigs'	Microbacterium gubbeenense DSM 15944	18.0	[14.9 - 21.5]	20.1	[17.9 - 22.5]	17.6	[15.0 - 20.6]	3.2
'AB.contigs'	Microbacterium atlanticum WY121	17.1	[14.1 - 20.7]	20.0	[17.8 - 22.5]	16.9	[14.3 - 19.9]	0.62
'AB.contigs'	Microbacterium luticocti DSM 19459	16.3	[13.3 - 19.8]	20.0	[17.8 - 22.4]	16.2	[13.7 - 19.1]	0.61
'AB.contigs'	Microbacterium flavum DSM 18909	16.5	[13.5 - 20.0]	20.0	[17.8 - 22.4]	16.4	[13.8 - 19.3]	0.99
'AB.contigs'	Microbacterium faecale CGMCC 1.15152	16.7	[13.7 - 20.2]	19.9	[17.7 - 22.3]	16.5	[14.0 - 19.5]	3.23
'AB.contigs'	Microbacterium telephonicum S2T63	18.2	[15.1 - 21.8]	19.8	[17.6 - 22.2]	17.7	[15.1 - 20.7]	0.39
'AB.contigs'	Microbacterium arborescens DSM 20754	17.8	[14.7 - 21.4]	19.7	[17.5 - 22.1]	17.4	[14.8 - 20.4]	2.03
'AB.contigs'	Microbacterium amylolyticum DSM 24221	15.2	[12.3 - 18.7]	19.1	[16.9 - 21.5]	15.3	[12.8 - 18.2]	6.83
'AB.contigs'	Microbacterium amylolyticum DSM 24221	15.2	[12.3 - 18.7]	19.1	[16.9 - 21.5]	15.3	[12.8 - 18.1]	6.83

Table S5. Ortho ANI values for *M. betulae* sp. nov. strain AB and the type strains of closely related *Microbacterium* species.

Genome B	Accession no	OrthoANIu value (%)	Genome length (bp)	Average aligned length (bp)	Genome AB^T coverage (%)	Genome B coverage (%)
M. barkeri	GCA_027921765.1_ASM2792176v1	82.52	3,472,080	1,305,900	35.91	37.61
M. oryzae	GCA_009735645.1_ASM973564v1	81.57	3,040,620	1,252,704	34.45	41.20
M. ulmi	GCA_013004565.1_ASM1300456v1	75.96	3,469,020	899,315	24.73	25.92
M. indicum	GCA_000422385.1_ASM42238v1	77.14	2,793,780	1,016,408	27.95	36.38
M. luticocti	GCA_000422405.1_ASM42240v1	75.11	3,103,860	796,595	21.91	25.66
M. gubbeenense	GCA_000422745.1_ASM42274v1	75.77	2,982,480	859,74	23.64	28.83
M. sediminis	CA_002741995.1_ASM274199v1	77.78	2,744,820	953,9	26.23	34.75
M. halophytorum	GCA_002970975.1_ASM297097v1	76.75	3,322,140	848,318	23.33	25.54
M. arborescens	GCA_003339645.1_ASM333964v1	75.08	3,389,460	971,104	26.71	28.65
M. telephonicum	GCA_003651225.1_ASM365122v1	75.76	3,115,080	922,306	25.36	29.61
M. sediminis	GCA_004564075.1_ASM456407v1	78.18	2,919,240	931,99	25.63	31.93
M. oryzae	GCA_009735645.1_ASM973564v1	81.57	3,040,620	1,252,704	34.45	41.20
M. karelineae	GCA_009745985.1_ASM974598v1	77.13	3,948,420	989,069	27.20	25,05
M. amylolyticum	GCA_011046975.1_ASM1104697v1	74.17	2,586,720	749,387	20.61	28.97
M. excoecariae	GCA_011326725.1_ASM1132672v1	76.92	2,710,140	938,91	25.82	34.64
M. faecale	GCA_014640975.1_ASM1464097v1	75.34	3,118,140	818,571	22.51	26.25
M. sorbitolivorans	GCA_014641475.1_ASM1464147v1	75.99	3,043,680	902,66	24.82	29.66
M. atlanticum	GCA_015277815.1_ASM1527781v1	75.70	3,712,800	915,862	25.19	24.67
M. flavum	GCA_018717645.1_ASM1871764v1	75.06	3,247,680	876,385	24,1	26,98
M. marinilacus	GCA_019753765.1_ASM1975376v1	77.38	3,871,920	1,170,838	32.20	30.24

Table S6. Clusters of orthologous groups (COG) of the *Microbacterium betulae* sp. nov. PCM 3040^{T} strain AB^{T}.

No.	**Functional category**	**Count**	**%**
1.	Carbohydrate metabolism	234	13.40
2.	Energy metabolism	62	3.55
3.	Lipid metabolism	36	2.06
4.	Nucleotide metabolism	78	4.47
5.	Amino acid metabolism	119	6.82
6.	Metabolism of other amino acids	18	1.03
7.	Glycan biosynthesis and metabolism	24	1.37
8.	Metabolism of cofactors and vitamins	81	4.64
9.	Metabolism of terpenoids and polyketides	18	1.03
10.	Biosynthesis of other secondary metabolites	6	0.34
11.	Xenobiotics biodegradation and metabolism	6	0.34
12.	Genetic information processing	163	9.34
13.	Environmental information processing	182	10.42
14.	Cellular processes	106	6.07
15.	Organismal systems	4	0.23
16.	Human diseases	0	0.00
17.	Protein families: metabolism	32	1.83
18.	Protein families: genetic information processing	172	9.85
19.	Protein families: signalling and cellular processes	198	11.34
20.	Unclassified: metabolism	90	5.15
21.	Unclassified: genetic information processing	19	1.09
22.	Unclassified: signalling and cellular processes	18	1.03
23.	Unclassified	80	4.58

Table S7. Secondary metabolite regions identified within the genome sequence of the *Microbacterium betulae* sp. nov. PCM 3040^T strain AB^T using the antiSMASH version 7.0 algorithm.

**Region**	**Type**	**From to**	**Most similar known cluster**
Region 1	type III polyketide synthase	695,816 736,559	5-acetyl-5,10-dihydrophenazine-1-carboxylic acid biosynthetic gene cluster from *Kitasatospora sp*. HKI 714
Region 2	beta lactone	2,255,899 2,283,627	beta lactone synthesis gene cluster from *Microbacterium oryzae* strain MB10T
Region 3	lasso peptide	2,323,250 2,345,797	lassopeptide synthase from *Kocuria indica* strain DE0236
Region 4	type III poly-ketide synthase	3,053,107 3,094,258	hierridin B biosynthetic gene cluster from *Cyanobium sp*. LEGE 06113
Region 5	terpene	3,136,897 3,157,772	carotenoid biosynthetic gene cluster from *Dietzia sp.* CQ4
Region 6	NI-siderophore	3,495,315 3,507,702	FW0622 biosynthetic gene cluster from *Verrucosispora sp.* FIM060022

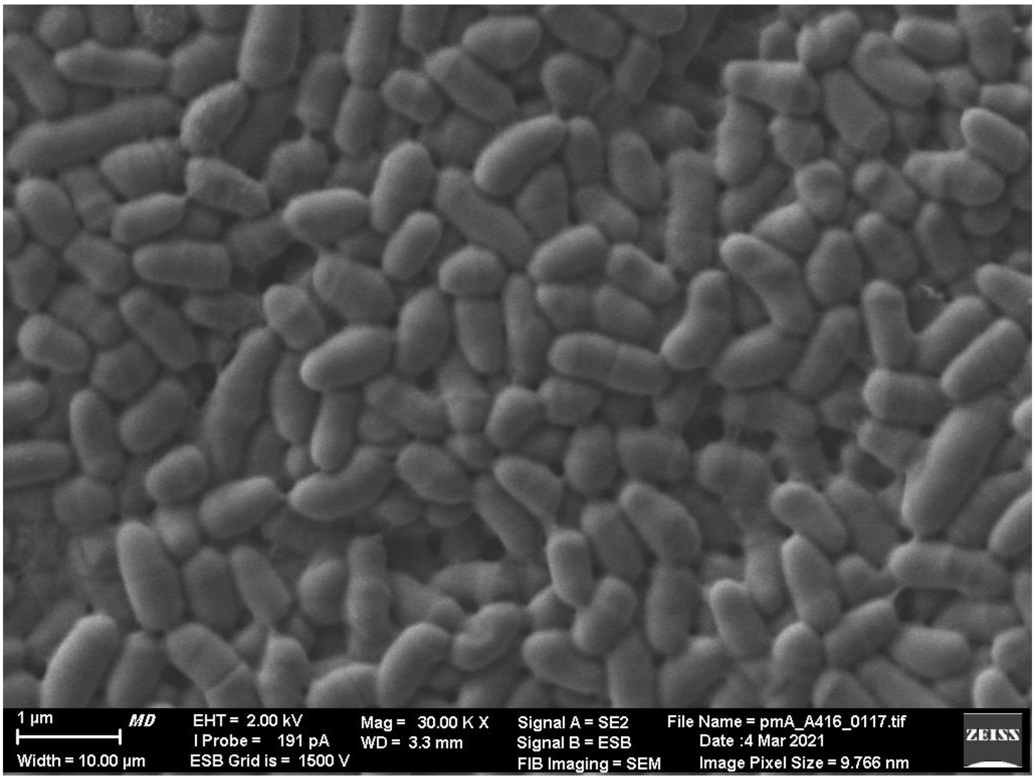

Figure S1. Scanning electron micrographs of *M. betulae* sp. nov. after growth on TSA agar at 28 °C for 48h. Bar, 1.0 µm.

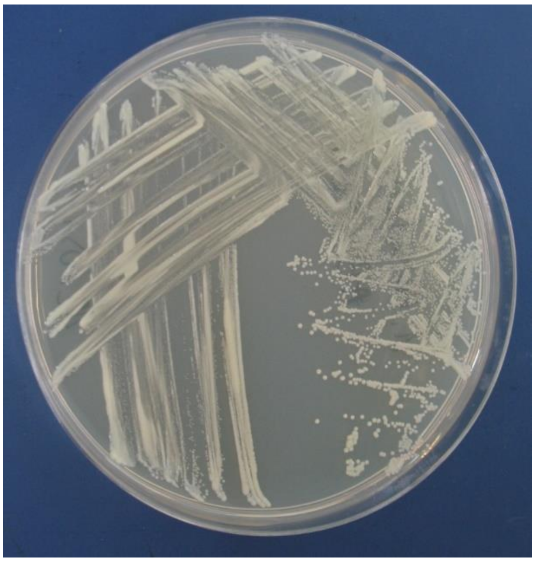

Figure S2. Colony morphology of *M. betulae* sp. nov., after growth on nutrient agar at 28 °C for 48h.

Chromatogram and Results

Injection Details

Injection Name:	**MK1**	*Run Time (min):*	**90,00**
Vial Number:	**RB3**	*Injection Volume:*	**3,00**
Injection Type:	**Unknown**	*Channel:*	**Emission_1**
Calibration Level:		*Wavelength:*	**n.a.**
Instrument Method:	**Metoda FLD 200 ulmin**	*Bandwidth:*	**n.a.**
Processing Method:	**Basic Quantitative**	*Dilution Factor:*	**1,0000**
Injection Date/Time:	**19.maj.23 12:20**	*Sample Weight:*	**1,0000**

Chromatogram

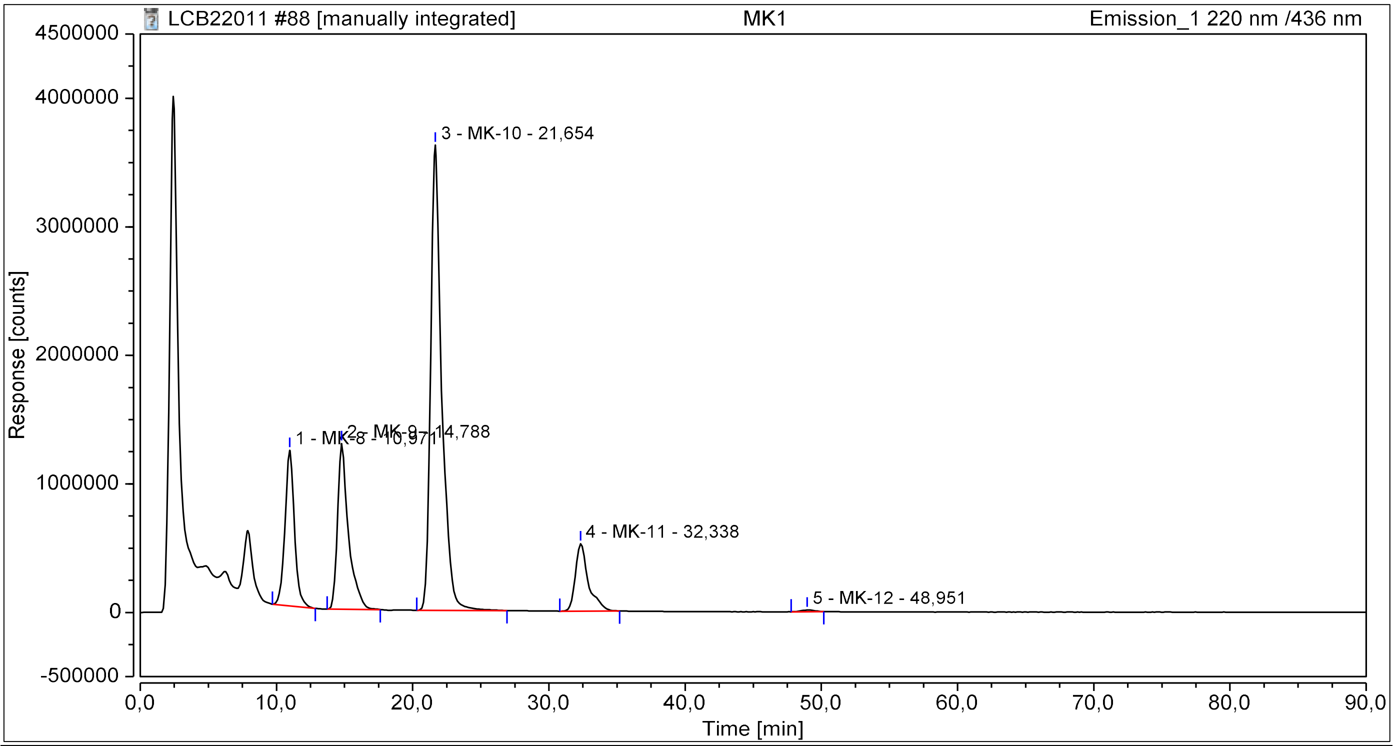

Integration Results

No.	Peak Name	Retention Time min	Area counts*min	Height counts	Relative Area %	Relative Height %	Amount
1	MK-8	10,971	975263,983	1211101,089	15,74	18,19	n.a.
2	MK-9	14,788	1205344,523	1289780,849	19,45	19,37	n.a.
3	MK-10	21,654	3421395,350	3619737,412	55,21	54,36	n.a.
4	MK-11	32,338	580443,018	525114,781	9,37	7,89	n.a.
5	MK-12	48,951	14763,648	13146,520	0,24	0,20	n.a.
**Total:**			**6197210,523**	**6658880,652**	**100,00**	**100,00**	

Figure S3. HPLC analysis of *M. betulae* sp. nov. showing the compositions of menaquinones.

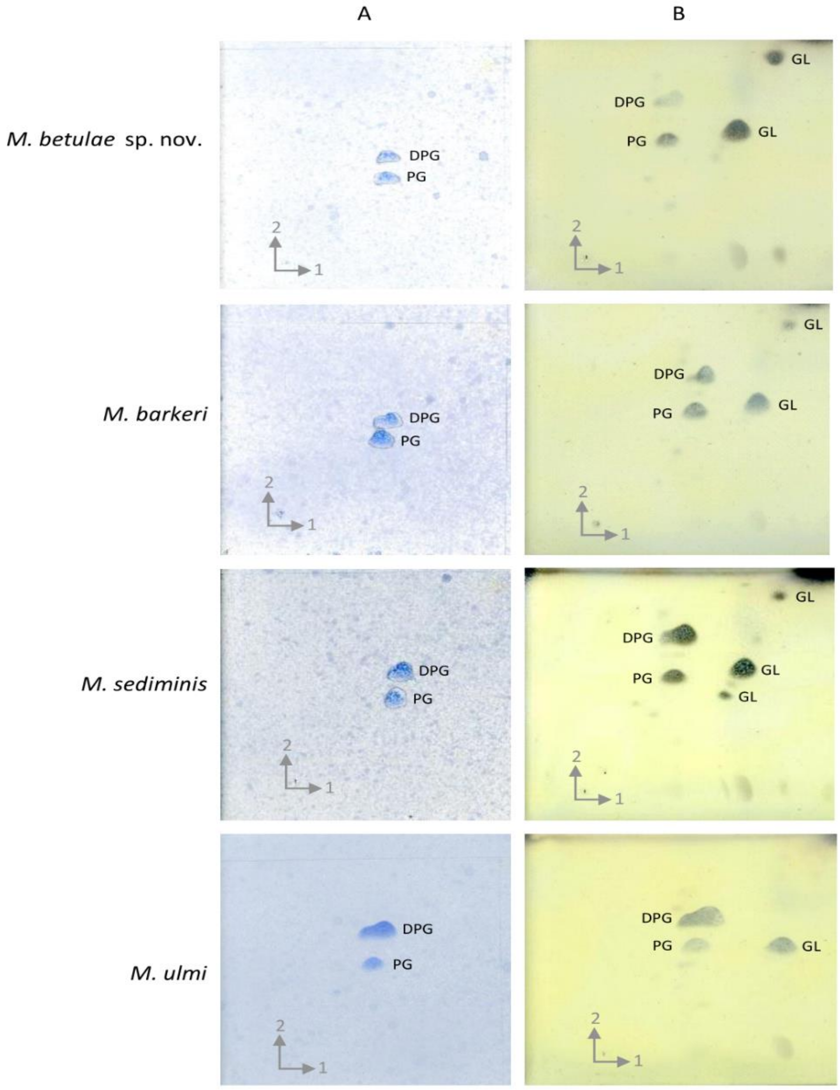

Figure S4. Two-dimensional thin layer chromatograms (2D-TLC) of polar lipids from *M. betulae* sp. nov., *M. barkeri*, *M. sediminis* and *M. ulmi*. Solvent systems: chloroform-methanol-water (65:25:4, v/v/v) was used in the first dimension (1), and chloroform-methanol-acetic acid-water (80:12:15:4, v/v/v/v) was used in the second dimension (2). Phospholipids (panel A) were detected using the Dittmer & Lester reagent, and total lipids (panel B) were detected using the phosphomolybdic acid reagent. Abbreviations: DPG, diphosphatidylglycerol; PG, phosphatidylglycerol; GL, glycolipid.

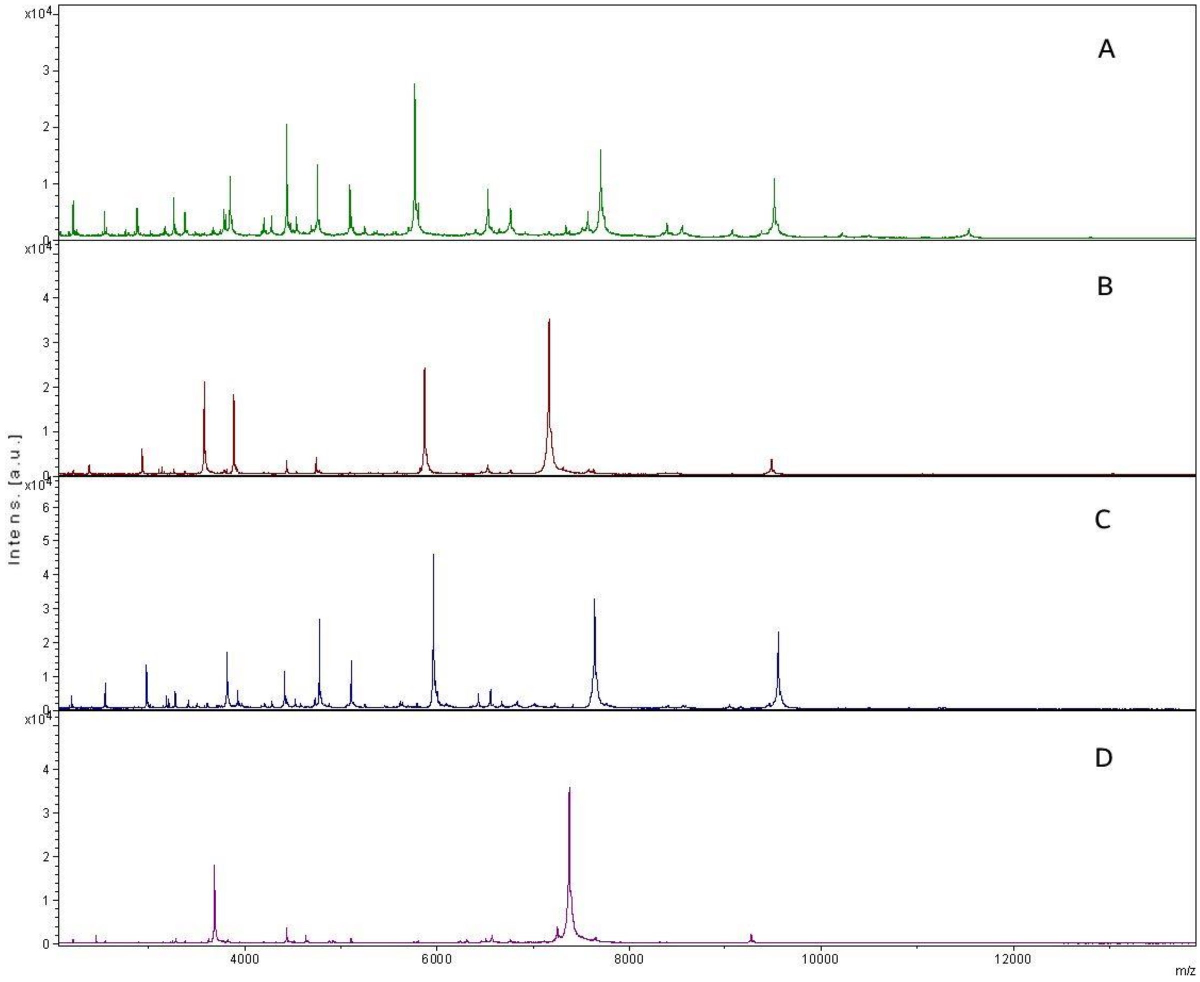

Figure S5. MALDI-TOF protein mass spectra of *M. barkeri* (A), *M. betulae* sp. nov. (B), *M. sediminis* (C), and *M. ulmi* (D).

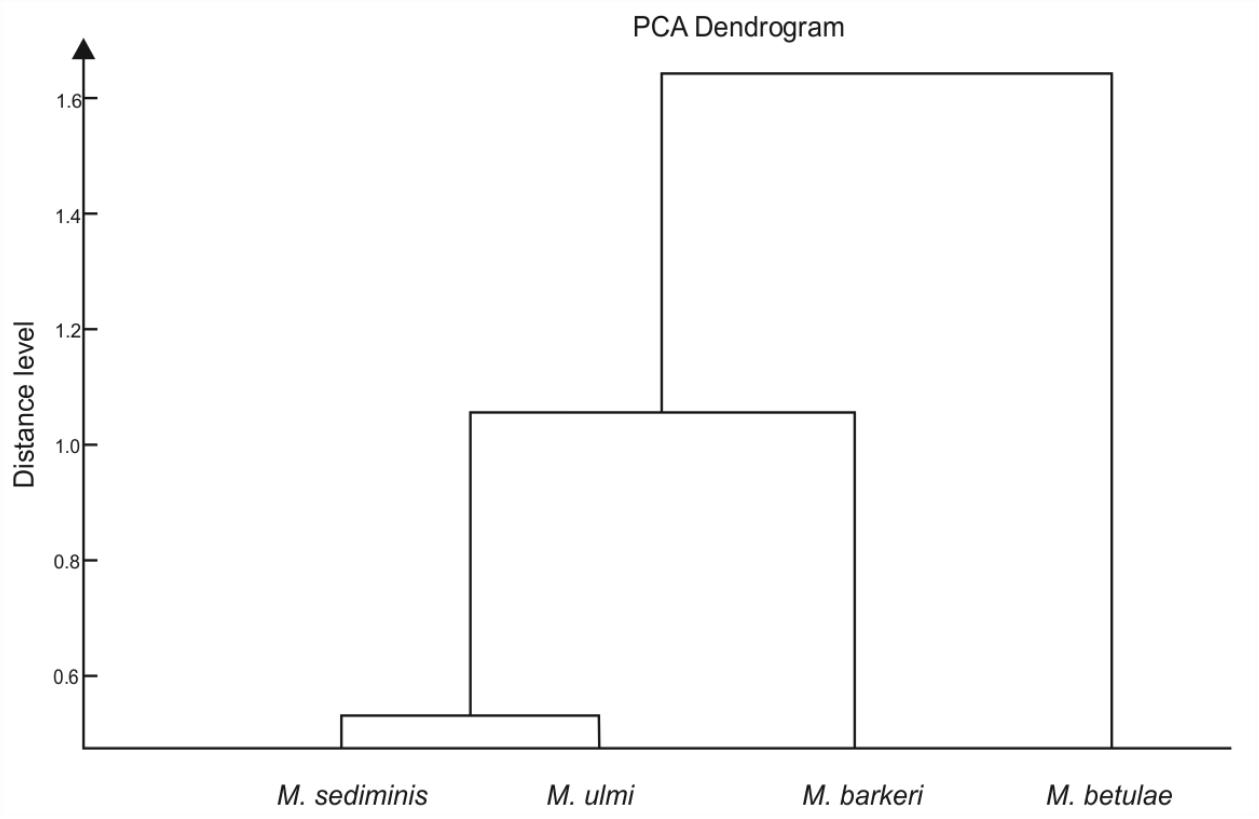

Figure S6. Cluster analysis of protein mass spectra of *M. sediminis*, *M. ulmi*, *M. barkeri* and *M.betulae* sp. nov. generated in MALDI Biotyper 3.0.

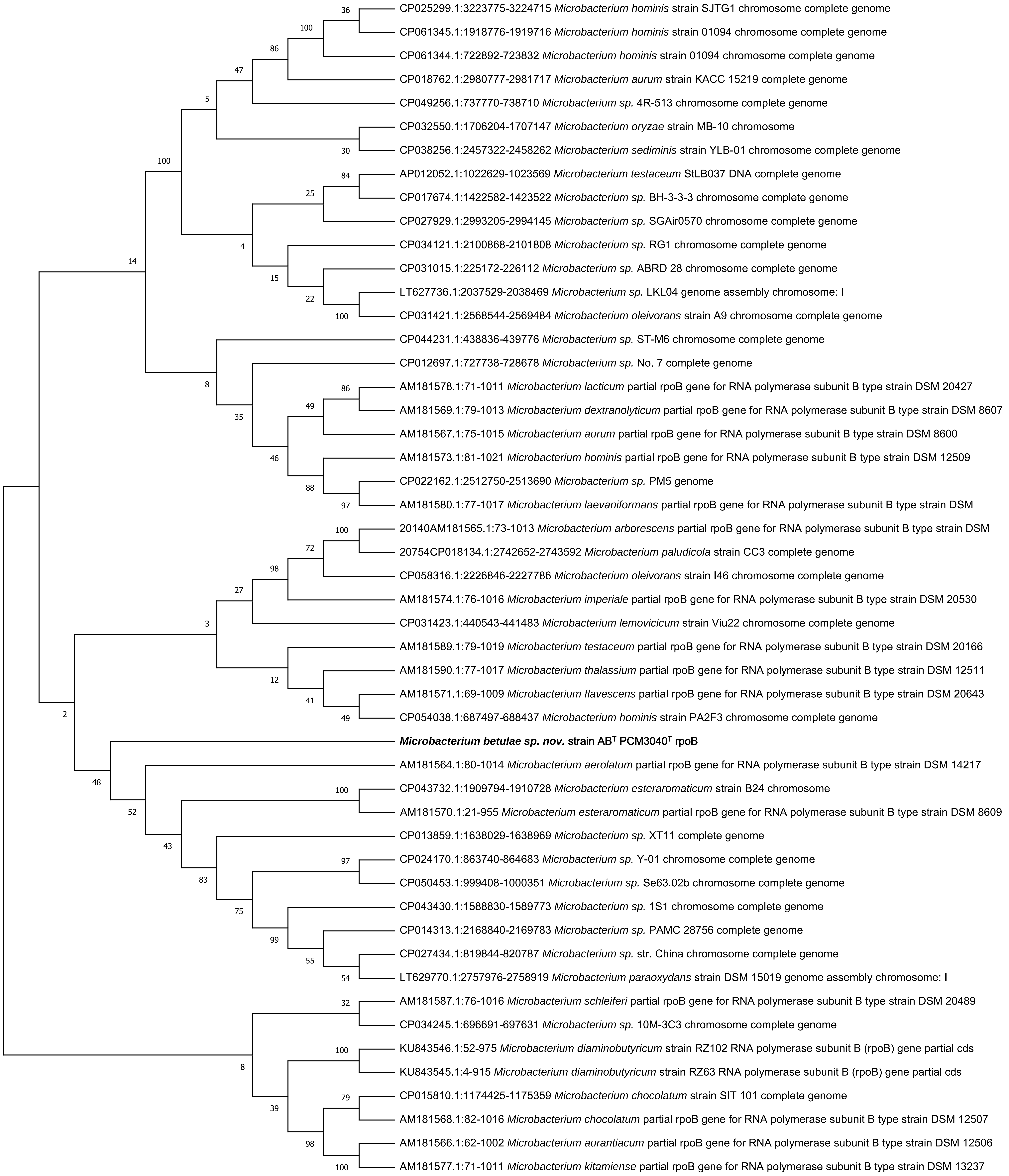

Figure S7. Maximum likelihood phylogenetic tree based on *rpoG* gene sequences showing the phylogenetic relationships of *M. betulae sp. nov.* to the closely related species of the genus *Microbacterium.* Bootstrap values from 500 replications are given at nodes. GeneBank accession numbers and ranges are given before the organism's name.

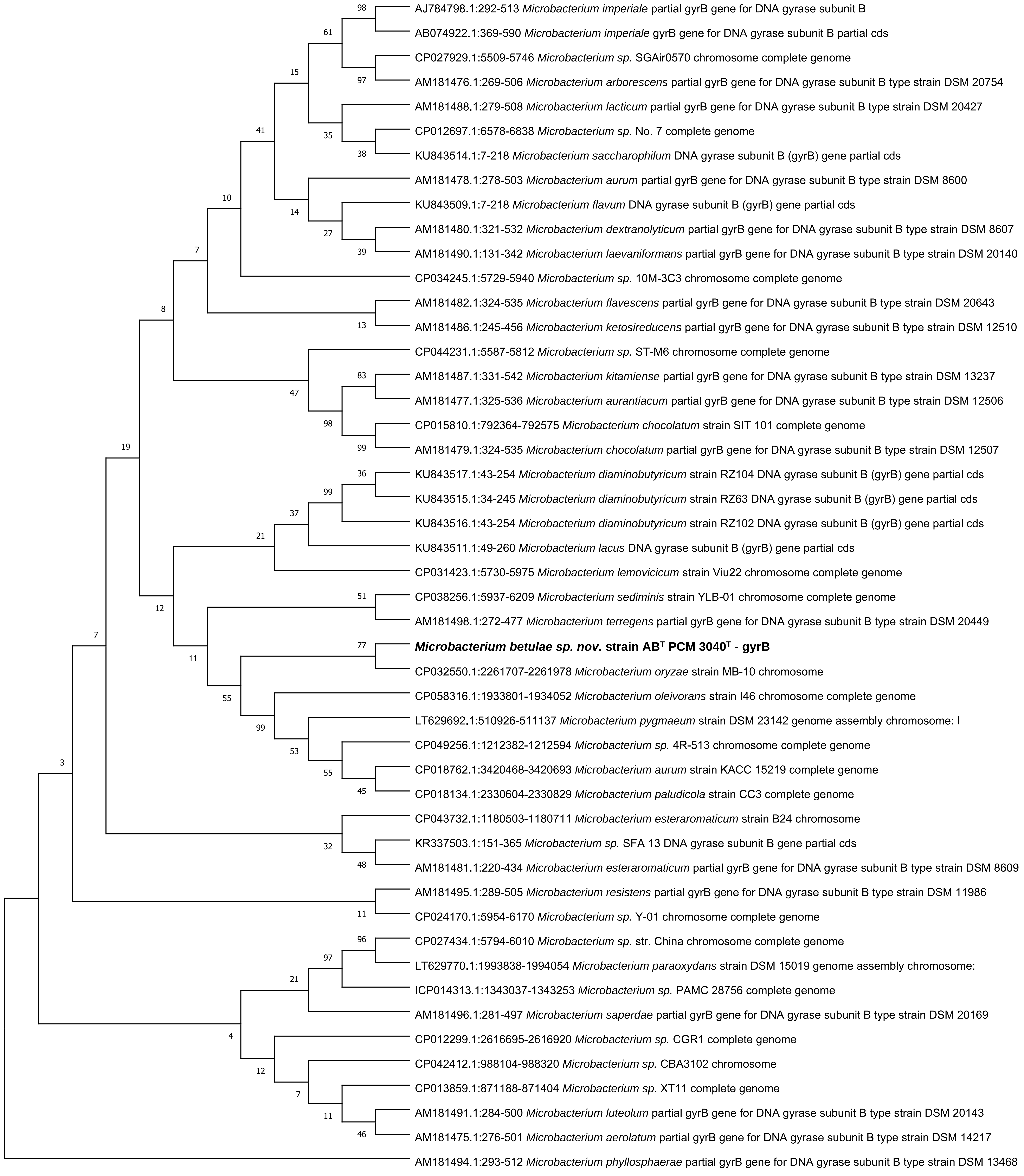

Figure S8. Maximum likelihood phylogenetic tree based on *gyrB* gene sequences showing the phylogenetic relationships of *M. betulae* sp. nov. to the closely related species of the genus *Microbacterium*. Bootstrap values from 500 replications are given at nodes. GeneBank accession numbers and ranges are given before the organism's name.
